# Stereoselective synthesis of *trans*-fused iridoid lactones and their identification in the parasitoid wasp *Alloxysta victrix*, Part I: Dihydronepetalactones

**DOI:** 10.3762/bjoc.8.140

**Published:** 2012-08-07

**Authors:** Nicole Zimmermann, Robert Hilgraf, Lutz Lehmann, Daniel Ibarra, Wittko Francke

**Affiliations:** 1 Department of Chemistry - Organic Chemistry, University of Hamburg, Martin-Luther-King-Platz 6, D-20146 Hamburg, Germany

**Keywords:** *Alloxysta victrix*, identification, iridoid, stereoselective synthesis, *trans*-fused dihydronepetalactone

## Abstract

Starting from the enantiomers of limonene, all eight stereoisomers of *trans*-fused dihydronepetalactones were synthesized. Key compounds were pure stereoisomers of 1-acetoxymethyl-2-methyl-5-(2-hydroxy-1-methylethyl)-1-cyclopentene. The stereogenic center of limonene was retained at position 4a of the target compounds and used to stereoselectively control the introduction of the other chiral centers during the synthesis. Basically, this approach could also be used for the synthesis of enantiomerically pure *trans*-fused iridomyrmecins. Using synthetic reference samples, the combination of enantioselective gas chromatography and mass spectrometry revealed that volatiles released by the endohyperparasitoid wasp *Alloxysta victrix* contain the enantiomerically pure *trans*-fused (4*R*,4a*R*,7*R*,7a*S*)-dihydronepetalactone as a minor component, showing an unusual (*R*)-configured stereogenic center at position 7.

## Introduction

**Figure 1:**
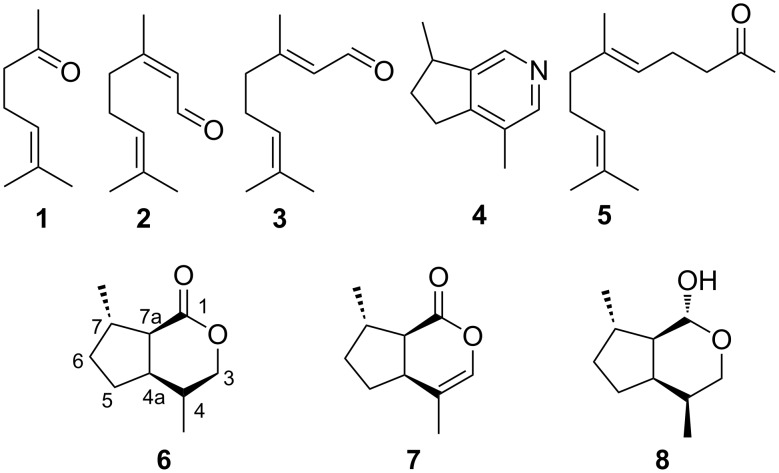
Terpenoids **1**–**5** present in *Alloxysta victrix* and *cis*-fused bicyclic iridoids known from other insects (**6**–**8**).

The endohyperparasitoid wasp *Alloxysta victrix* is part of the tetratrophic system of oat plants (*Avena sativa*), grain aphids (*Sitobion avenae*), primary parasitoids (*Aphidius uzbekistanicus*) and hyperparasitoids (*Alloxysta victrix*). Chemical communication by volatile signals is considered to play a major role in interactions between these trophic levels, and some semiochemicals of the lower trophic levels such as oat plant and grain aphid have been identified [2–4]. However, there is nearly no information about the intra- and interspecific signaling pathways between primary parasitoids and hyperparasitoids. In order to gain further information about the chemical structures and the biological significance of corresponding signals, we examined the volatile components of pentane extracts from dissected heads as well as headspace volatiles of *Alloxysta victrix* by coupled gas chromatography/mass spectrometry (GC/MS). Figure 1 shows one major component and several trace components which could be identified as 6-methyl-5-hepten-2-one (**1**), neral (**2**), geranial (**3**), actinidine (**4**), and geranylacetone (**5**). Bioassays revealed the main compound **1** to be repellent to the aphid-parasitoid, *Aphidius*, by warning the primary parasitoid of the presence of the hyperparasitoid [5]. The prenyl-homologue of **1**, geranylacetone (**5**), seems to be a component of the sex pheromone of *Alloxysta victrix* [6]. In addition, GC/MS of cephalic secretions of both sexes showed a minor component **X**, the mass spectrum of which suggested it to be a *trans*-fused dihydronepetalactone. Since no synthetic reference compounds were available, we had to synthesize all eight *trans*-fused dihydronepetalactones to unambiguously identify the natural product **X**. The realization of this task is the subject of the present paper.

## Results and Discussion

**Figure 2:**
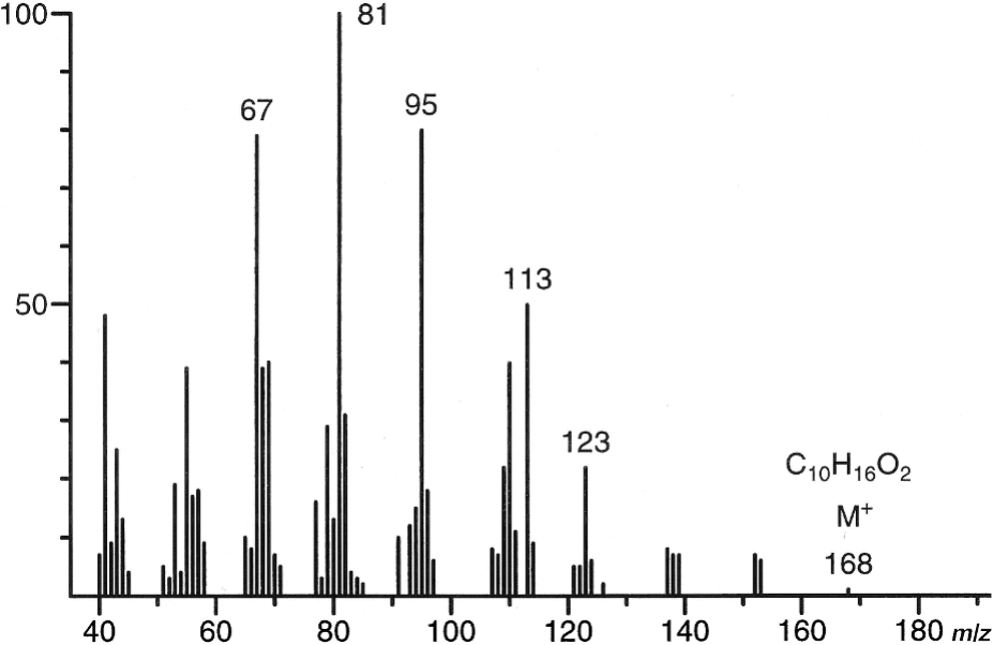
70 eV EI-mass spectrum of the iridoid **X**, a component of the volatile secretions of the parasitoid wasp *Alloxysta victrix*.

Apart from a couple of known acyclic terpenoids (Figure 1), analysis by gas chromatography coupled with mass spectrometry (GC/MS) revealed the presence of an unknown minor component **X** in both sexes of *Alloxysta victrix*. Chemical ionization analysis (GC/CIMS) showed the molecular mass of the compound to be M^+^ = 168, while high resolution mass spectrometry (GC/HRMS) proved its atomic composition to be C_10_H_16_O_2_, suggesting an oxygenated monoterpene as the target structure. The fragmentation pattern, exhibited in the 70 eV EI-mass spectrum (Figure 2), showed some similarities to that of the known *cis*-fused dihydronepetalactone (**6**) [7], however, differences in relative abundances of fragment ions pointed to a *trans*-fused dihydronepetalactone as the target structure [8]. In the mass spectrum of the *cis*-fused compound *m*/*z* 67 and *m*/*z* 95 were of similarly low intensity (30%), while in that of the unknown natural product **X** the two fragments were highly abundant (80%). The most striking differences in the spectra were pronounced signals for the molecular ion M^+^ = 168 and M^+^ − 15 (70% and 40%, respectively) for the *cis*-fused dihydronepetalactone whilst in the spectrum of **X** the two signals were of only low abundance (Figure 2).

Dihydronepetalactones are derivatives of nepetalactone (**7**) which was first isolated by Mc Elvain in 1941 from the essential oil of catmint, *Nepeta cataria* (Figure 1) [9]. Relative configurations of *cis*-fused nepetalactones and some related derivatives have been investigated [10–11]. Nepetalactone and *cis*- as well as *trans*-fused dihydronepetalactones have been isolated from the leaves and galls of the plant *Actinidia polygama* [8]. In addition, dihydronepetalactones are components of the defensive secretions of some ant species [12], while nepetalactone and the corresponding lactol showing (1*R*)-configuration have been identified as pheromones of aphids [13–14]. (1*R*,4*S*,4a*R*,7*S*,7a*R*)-Dihydronepetalactol (**8**) was characterized as a semiochemical for lacewings [15].

**Figure 3:**
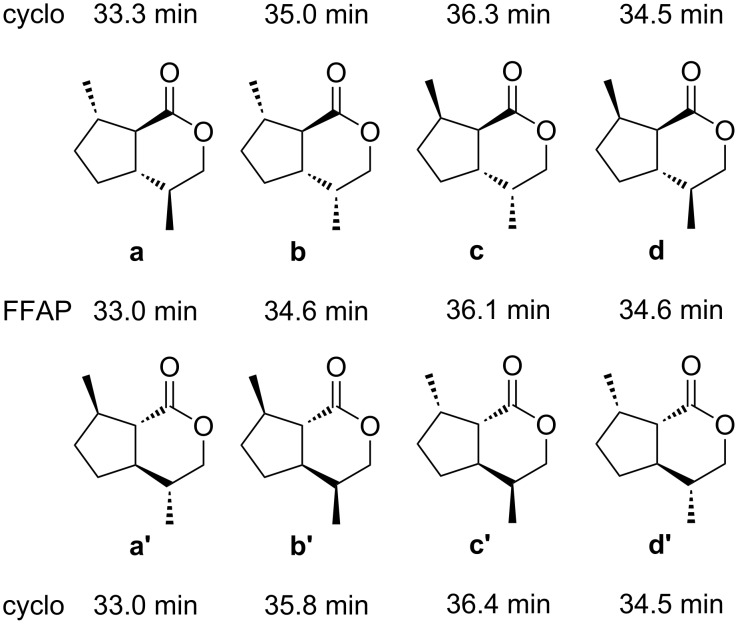
Structures and gas chromatographic retention times of *trans*-fused dihydronepetalactones on a conventional FFAP column (FFAP) and on an enantioselective cyclodextrin column (cyclo). For experimental details see Supporting Information File 1. The racemates **b**/**b'** and **d**/**d'**, which coeluted on FFAP, could be separated from each other on DB5 (data not shown).

The dihydronepetalactone skeleton shows four contiguous stereogenic centers, giving rise to eight *trans*-fused stereoisomers **a**–**d** and the corresponding enantiomers **a'**–**d'** (Figure 3).

Whilst several stereoselective syntheses of the relatively widespread and well known *cis*-fused nepetalactone and its dihydro derivatives have been carried out [16–19], only very few approaches specifically aiming at the synthesis of *trans*-fused iridoid lactones have been published. Starting from (*S*)-pulegone (**9**) or its enantiomer, Wolinsky [20–21] described a route to this group of iridoids that can be applied to synthesize pure stereoisomers of dihydronepetalactones as well as the structurally related iridomyrmecins, another class of iridoids. However, Wolinsky’s method suffers from several major disadvantages such as high costs of (*S*)-pulegone and difficult separations of diastereomeric mixtures. Therefore, as an alternative, we present a novel stereoselective route to *trans*-fused dihydronepetalactones starting from pure, cheaply available enantiomers of limonene.

### Route to *trans*-fused dihydronepetalactones **a** and **b** starting from (*S*)-pulegone

**Scheme 1:**
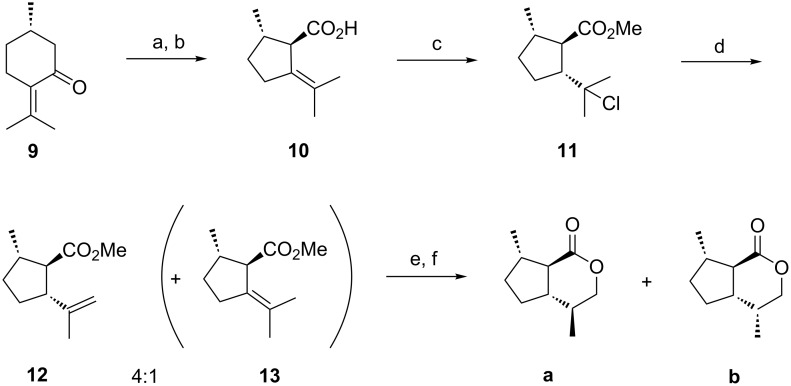
Route from (*S*)-pulegone to the mixture of dihydronepetalactones **a** and **b**, consequently following Wolinsky's approach [23]. Reaction conditions: a) Br_2_, HOAc, 0 °C; b) KOH, H_2_O, reflux (15%, 2 steps); c) MeOH/HCl, rt, 96 h (82%); d) 2,6-lutidine, reflux, 72 h (75% of **12** + **13**); e) BH_3_·SMe_2_, 0 °C, THF, NaOH, H_2_O_2_ (86% mixture of diastereomers); f) *p*-TsOH, toluene, reflux (17% of a + b).

For comparison, the synthesis of **a** and **b** was carried out following Wolinsky’s approach: (*S*)-Pulegone (**9**) was transformed to *trans*-pulegenic acid **10** via bromination, Favorskii rearrangement, and subsequent elimination (Scheme 1). Stereoselective addition of hydrochloric acid afforded the chloride **11**, and subsequent elimination of hydrochloric acid gave a mixture of the methyl esters **12** and **13** (methyl *trans*-pulegenate) [20–22] which could be separated by chromatography on silica gel. Hydroboration and lactonization of **12** furnished a mixture of the C4-epimers **a** and **b** that once again needed to be separated by chromatography on silica gel [23].

**Figure 4:**
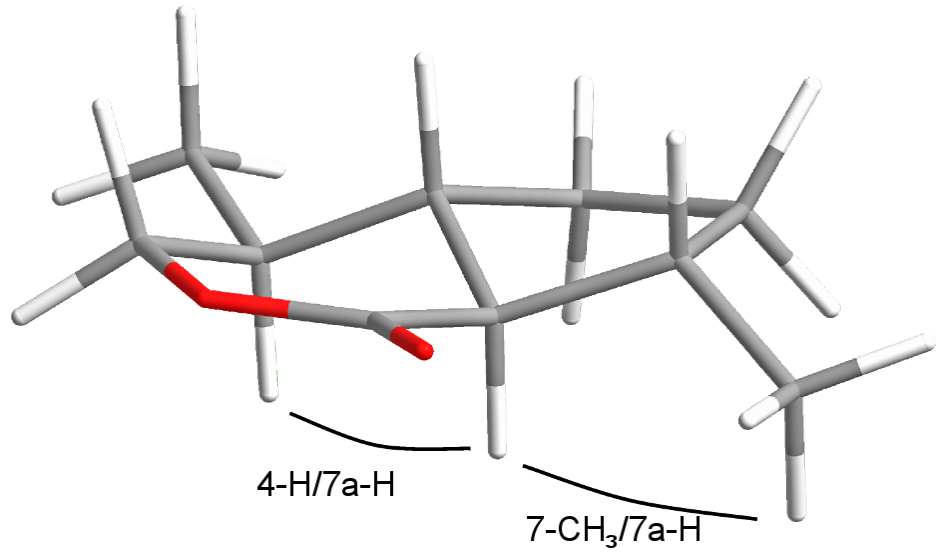
Configuration of the dihydronepetalactone **a**.

Analytical data of the first eluting component **a** were in accordance with those reported in the literature [24]. The same sequence starting from (*R*)-pulegone yielded a mixture of diastereomers **a'** and **b'**. The relative configuration of **a** at C4 was assigned according to NOESY experiments. Decisive NO-effects were found between the protons 4-H and 7a-H as well as between 7a-H and 7-CH_3_ (Figure 4).

Basically, the sequence developed by Wolinsky could also provide access to the diastereomers **c** and **d** (and their enantiomers) if *trans*-pulegenic acid (**10**) would be replaced by *cis*-pulegenic acid. A mixture of the latter and its *trans-*isomer (60:40) can be obtained by using a different base in the Favorskii-rearrangement step [22], again requiring a difficult chromatographic separation. Furthermore, this multistep route has several major disadvantages: The formation of mixtures of epimers entails to separations at several stages which have proven to be problematic. Moreover, several reaction steps afford unsatisfactory yields [23]. In addition, one of the main disadvantages is the fact that (*S*)-pulegone (*S*-**9**) is a highly expensive starting material for the synthesis of four of the eight *trans*-fused dihydronepetalactones. That excludes this route for the synthesis of larger amounts.

### Route to stereochemically pure *trans*-fused dihydronepetalactones from (*R*)-limonene

**Figure 5:**
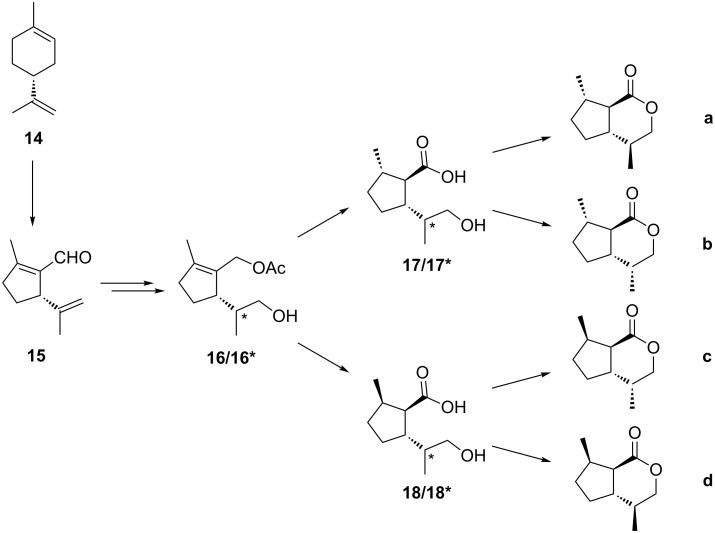
Route to stereochemically pure *trans*-fused dihydronepetalactones **a**–**d** from (*R*)-limonene.

Due to the shortcomings of the route described above, we designed an improved strategy towards *trans*-fused dihydronepetalactones. Starting from 1-formyl-2-methyl-5-(1-methylethenyl)-1-cyclopentene (**15**) as the key intermediate, the stereoselective synthesis of all eight stereoisomers could be achieved (Figure 5). The aldehyde **15** could be readily prepared from commercially available pure and cheap (*R*)-limonene (**14**) [25–27]. Non-selective hydroboration of the double bond in the side chain of **15** would yield a pair of diastereomers **16**/16* which would have to be separated. However, we expected that the chiral center at C5 would cause stereocontrol by forcing the reaction to proceed through the sterically least hindered transition state. We envisioned that stereoselective hydrogenation of the endocyclic double bond of the key intermediate **16** (and its diastereomer 16*) in either a “*syn*”- or “*anti*”-fashion could yield two pairs of diastereomeric hydroxy carboxylic acids 17/17* or 18/18* after some simple functional group transformations. These hydroxy acids would then yield the desired *trans*-fused dihydronepetalactones **a**–**d** during a final lactonization step.

Starting from cheap and pure (*S*)-limonene (**14'**), the corresponding *trans*-fused dihydronepetalactones **a'**–**d'** could be synthesized in the same way, showing our novel route to be a versatile alternative to Wolinsky’s sequence [20–21]. In contrast to the latter, which fixed the stereogenic center of pulegone at position 7 of the final dihydronepetalactone, in our route the stereogenic center of limonene is retained at position 4a of the target compound and used for the stereoselective introduction of additional chiral centers.

### Synthesis of the key intermediate **16**

**Scheme 2:**
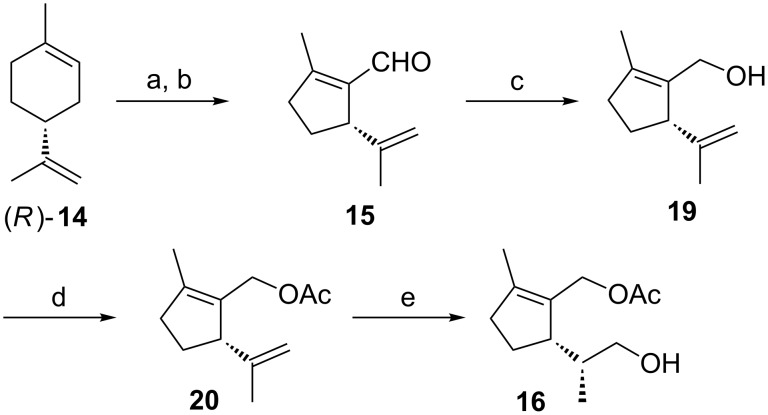
Synthesis of the key compound **16**. Reaction conditions: a) O_3_, MeOH, −50 °C (86%); b) AcOH, piperidine, C_6_H_6_, reflux (85%); c) LiAlH_4_, Et_2_O (82%); d) Ac_2_O, pyridine, rt (91%); e) B_2_H_6_, 2-methyl-2-butene, 0 °C, THF, NaOH, H_2_O_2_ (66%).

The synthesis of the key intermediate **16** – which shows two differentiated primary alcohol functions – started from enantiomerically pure (*R*)-limonene (**14**, Scheme 2). Ozonolysis followed by reductive workup with dimethyl sulfide produced (3*R*)-3-(1-methylethenyl-6-oxoheptanal), which yielded the formyl cyclopentene **15** upon intramolecular aldol condensation [25–27]. Subsequently, the aldehyde **15** was reduced to the allylic alcohol **19** with LiAlH_4_ and converted into the acetate **20** [28]. Hydroboration of **20** using disiamylborane proceeded with high stereoselectivity affording **16** as a single stereoisomer [17,28]. Similar results of highly stereoselective hydroborations of structurally related chiral cyclopentene derivatives have been reported [20–21].

### Synthesis of *trans*,*trans*-dihydronepetalactone **b**

**Scheme 3:**
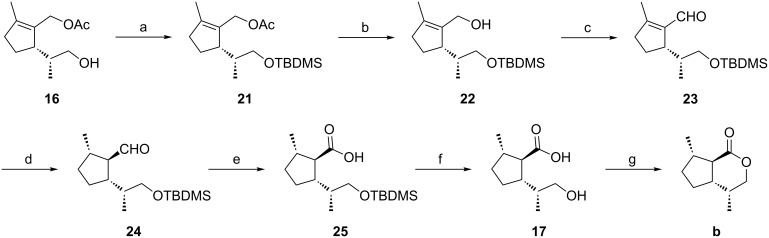
Synthesis of *trans*,*trans*-substituted dihydronepetalactone **b**. Reaction conditions: a) TBDMSCl, imidazole, DMF, 0 °C (99%); b) KOH, MeOH, 0 °C (87%); c) PDC, MS, CH_2_Cl_2_, 0 °C to rt (61%); d) ammonium formate, Pd/C, MeOH, reflux (48%); e) KMnO_4_, *t*-BuOH, pH 4.5, 0 °C (22%); f) TBAF, THF (100%); g) DCC, DMAP, CH_2_Cl_2_ (66%).

To install a *trans*,*trans*-configuration between the substituents at C5-C1 and C1-C2 of the cyclopentane backbone – which would later reflect the *trans,trans* relationship between substituents at C7-C7a and at C7a-C4a of the dihydronepetalactones **a** and **b** – a formal “*anti*”-addition of hydrogen to the cyclopentene **16** had to be carried out (Scheme 3). Usually, both homogeneous and heterogeneous catalytic hydrogenation reactions proceed via “*syn*”-addition of hydrogen to olefinic double bonds. Only subsequent isomerization processes may lead to a formal “*anti*”-addition. To obtain a suitable precursor, which, due to enolization of the hydrogenation product, might allow this formal “*anti*”-addition of hydrogen, the key intermediate **16** was transformed to the aldehyde **23**.

In the course of this short sequence, the free hydroxy group of **16** was protected as the TBDMS ether to yield **21** which afforded the mono-protected diol **22** after treatment with KOH in methanol. Subsequently, **22** was oxidized with pyridinium dichromate to give aldehyde **23**.

**Figure 6:**
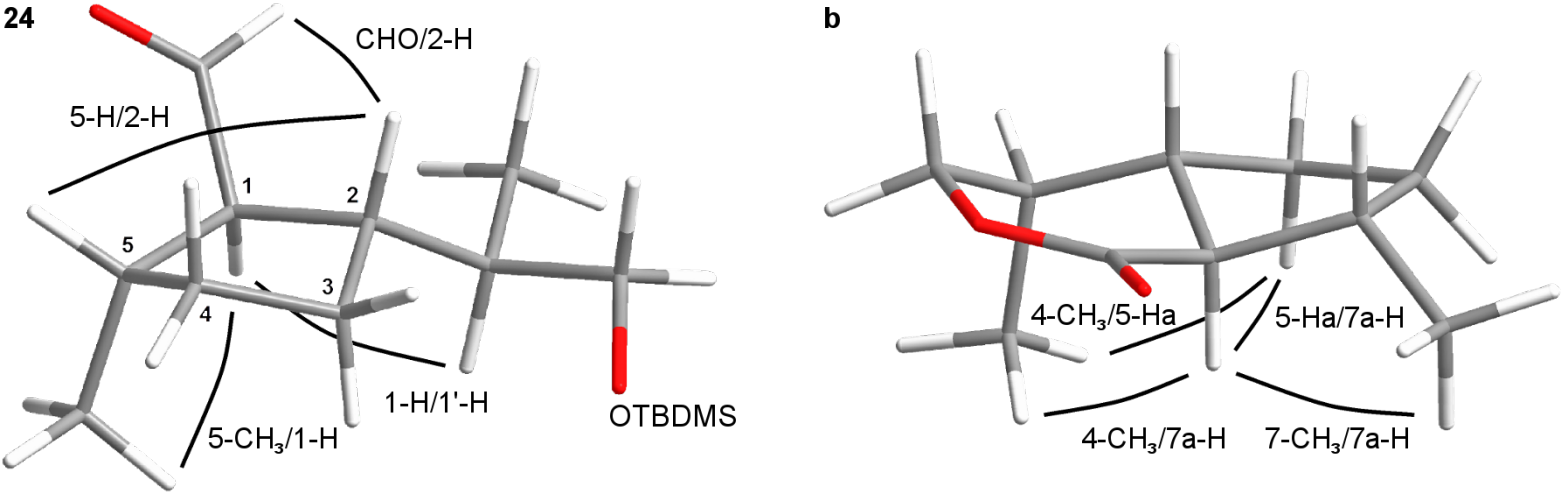
Configurations of compound **24** and the dihydronepetalactone **b**.

We expected that catalytic hydrogenation of the trisubstituted cylopentene **23** with a heterogeneous catalyst would preferentially take place from the sterically less hindered side of the molecule. This would lead to an all-*cis* configured hydrogenation product which would endure considerable steric strain. Due to the CH-acidity at the α-position of the formyl group, epimerization of the all-*trans* product **24** under acidic or basic conditions could be expected. Lange et al. reported the catalytic hydrogenation of a structurally close analogue, (5*R*)-1-formyl-2-methyl-5-isopropylcyclopent-1-ene, over Pd/C (10%) to give a 9:1 mixture of the all-*cis* versus the all-*trans* product [29]. In our case, the application of Lange’s method to the aldehyde **23** led to the formation of a 3:1 mixture of the all-*cis* versus the all-*trans* epimer. Subsequent treatment with sodium methoxide in MeOH at rt for 20 h completely shifted the equilibrium to the thermodynamically more stable all-*trans* product **24**. Unfortunately, these results could not be reproduced on larger reaction scales (>5 mmol). After screening of a variety of other hydrogenation conditions, we found the hydrogenation of **23** with ammonium formate over palladium on carbon (10%) to be the method of choice [30]. Using this approach, the all-*trans* aldehyde **24** was almost exclusively formed. The presence of ammonium formate in the reaction mixture probably leads to an “in-situ” epimerization at C2 from the kinetically formed all-*cis* to the thermodynamically more stable all-*trans* product. Relative configurations of all substituents of compound **24** were confirmed by NOESY experiments (Figure 6). Strong NO-effects were found between the protons 5-CH_3_ and 1-H, 1-H and 1’H (the proton at C1 of the side chain), 5-H and 2-H as well as between protons of 1-CHO and 2-H which is in line with a *trans,trans*-configuration (using the nomenclature described above).

Starting from **24**, the *trans*,*trans*-dihydronepetalactone **b** was synthesized in three subsequent steps (Scheme 3). First, oxidation of the aldehyde group with potassium permanganate in the presence of a phosphate buffer (pH 4.5) afforded the carboxylic acid **25** without epimerization at C1 [23,31]. Subsequent deprotection of the TBDMS ether with tetrabutylammonium fluoride (TBAF) yielded **17**, and lactonization with *N*,*N*-dicyclohexylcarbodiimide (DCC) and 4-dimethylaminopyridine (DMAP) in dichloromethane afforded the *trans*,*trans*-dihydronepetalactone **b**.

The relative configuration of the *trans*,*trans*-dihydronepetalactone **b** was confirmed by NOESY experiments. Decisive NO-effects could be observed between 4-CH_3_ and 7a-H as well as between 4-CH_3_ and 5-Ha, and furthermore, between 5-Ha and 7a-H as well as between 7a-H and 7-CH_3_ (Figure 6). The enantiomer **b'** was synthesized via the same route starting from (*S*)-limonene. Analytical data of compound **b'** were identical to those which were obtained of **b** when Wolinky’s route was followed (see above).

### Synthesis of *cis*,*trans*-fused dihydronepetalactone **c**

**Scheme 4:**
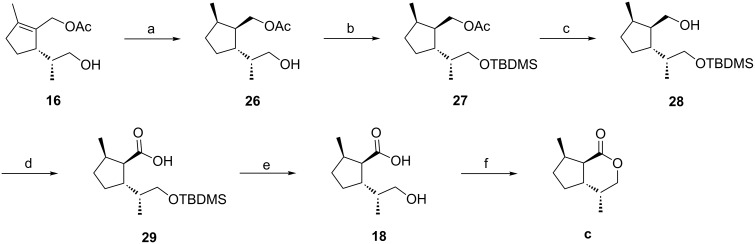
Synthesis of *cis*,*trans*-substituted dihydronepetalactone **c**. Reaction conditions: a) Crabtree's catalyst [Ir(cod)PCy_3_(py)]PF_6_ (11 mol %), 1 bar H_2_, CH_2_Cl_2_, rt (81%); b) TBDMSCl, imidazole, DMF, 0 °C (100%); c) KOH, MeOH, rt (93%); d) RuCl_3_·3H_2_O (2 mol %), NaIO_4_, CCl_4_, CH_3_CN, phosphate buffer (pH 7), rt (81%); e) HF, CH_3_CN, rt (100%); f) DCC, DMAP, CH_2_Cl_2_ (66%).

For the synthesis of the *cis*,*trans* dihydronepetalactone **c**, a *cis*,*trans*-configuration between the substituents at C5-C1 and C1-C2 of the cyclopentane backbone needed to be established. With acetate **16** as the key intermediate, a stereoselective “*syn*”-addition of hydrogen from the same side as the (*R*)-configured side chain at C5 would provide the desired stereochemical outcome of the hydrogenation reaction (Scheme 4). We expected the free hydroxy group of **16** to coordinate to an appropriate homogenous hydrogenation catalyst, controlling the stereochemical course of the hydrogen transfer from the same side as the side chain at C5 through chelation. We chose Crabtree’s iridium catalyst ([Ir(cod)PCy_3_(py)]PF_6_) which has been reported to furnish excellent facial selectivities during directed hydrogenations of cyclic olefins [32–34].

Hydrogenation of acetate **16** in the presence of 11 mol % of Crabtree’s catalyst under 1 bar hydrogen pressure for 1.5 h yielded the desired product **26** as a single diastereomer. Alternative hydrogenation methods using optically active catalysts failed. In one case we investigated the hydrogenation of the endocylic double bond of the allylic alcohol **19** (Scheme 2) with one of Noyori’s ruthenium BINAP catalysts ([Ru((*S*)-BINAP)](OAc)_2_) [35–36] but reduction occurred only at the side chain.

**Figure 7:**
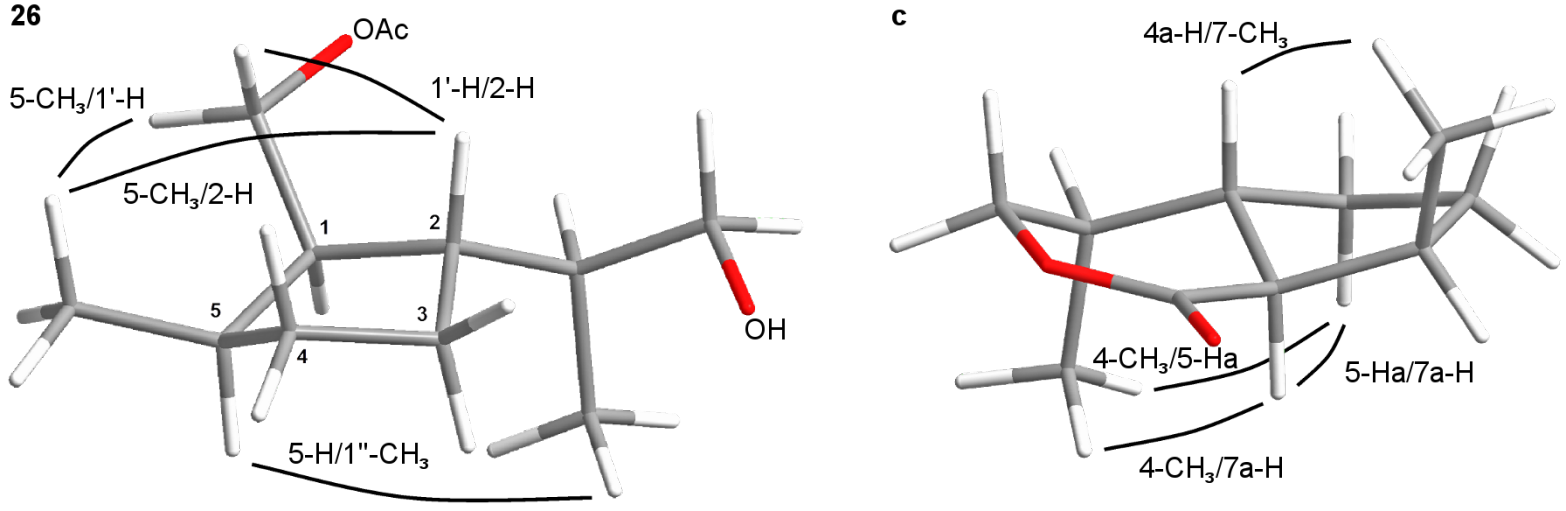
Configurations of compound **26** and the dihydronepetalactone **c**.

Relative configurations of all substituents of the acetate **26** were confirmed by NOESY experiments (Figure 7). Strong NO-effects were observed between the protons 5-CH_3_ and 1’-H (protons of the acetoxymethyl group at C1), 5-CH_3_ and 2-H, 1’-H and 2-H as well as 5-H and 1’’-CH_3_ (protons of the methyl group at C1 of the side chain) which is in line with a *cis*,*trans*-configuration (using the nomenclature described above).

Starting from **26**, the synthesis of the *cis,trans-*dihydronepetalactone **c** was completed in five subsequent steps (Scheme 4). First, the free hydroxy group of **26** was protected as the TBDMS ether to yield **27**. Then, the acetate group was removed with methanolic KOH to afford the alcohol **28**. Careful oxidation of the primary alcohol function [37–38] with ruthenium(III) chloride and sodium periodate in a biphasic mixture of carbon tetrachloride, acetonitrile, and phosphate buffer (pH 7) produced the carboxylic acid **29** without epimerization at C1. After removal of the TBDMS protecting group with HF in acetonitrile, the hydroxy acid **18** was lactonized in the presence of DCC and catalytic amounts of DMAP in dichloromethane at rt to afford *cis,trans-*dihydronepetalactone **c**. Its enantiomer **c’** was synthesized from enantiomerically pure (*S*)-limonene, following the same route. The relative configuration of **c** was confirmed by NOESY experiments (Figure 7). Decisive NO-effects could be observed between 4a-H and 7-CH_3_ as well as between 4-CH_3_ and 7a-H, and furthermore, between 4-CH_3_ and 5-Ha as well as between 5-Ha and 7a-H.

### Synthesis of a mixture of *trans*-fused dihydronepetalactones **c** and **d**

**Scheme 5:**
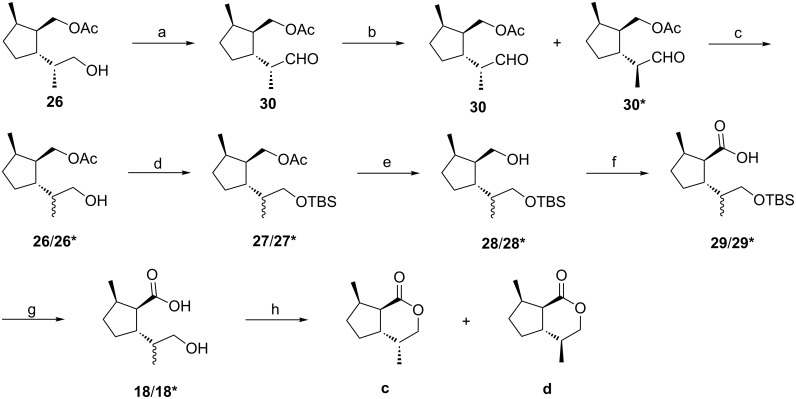
Synthesis of a 2:3 mixture of dihydronepetalactones **c** and **d**. Reaction conditions: a) (COCl)_2_, DMSO, CH_2_Cl_2_, −70 °C to 0 °C (71%); b) *p*-TsOH, benzene, reflux (96%); c) NaBH_4_, MeOH, −20 °C; d) TBDMSCl, imidazole, DMF, 0 °C (78%) (over two steps); e) KOH, MeOH, rt (94%); f) RuCl_3_·3H_2_O (2 mol %), NaIO_4_, CCl_4_, CH_3_CN, phosphate buffer (pH 7), rt (69%); g) HF, CH_3_CN, rt (98%); h) DCC, DMAP, CH_2_Cl_2_ (62%).

The stereogenic center at C1’ of the acetate **26** keeps (*R*)-configuration which resulted from highly stereoselective hydroboration of the acetate **20** to yield the key intermediate **16** as shown above (Scheme 2). For the synthesis of the *cis,trans-*dihydronepetalactone **d**, this stereocenter needed to be isomerized to keep the (4*S*)-configuration in the final product (Scheme 5). To achieve the required inversion, the acetate **26** was oxidized to the aldehyde **30**, which could be epimerized using *p*-toluenesulfonic acid in benzene under reflux conditions to provide a 2:3 mixture of the desired aldehydes **30** and its epimer 30*. Subsequent steps were carried out with the mixture of diastereomers. Reaction of **30**/30* with sodium borohydride at −20 °C reduced the aldehyde function to yield a mixture of the diastereomers **26**/26*. The following sequence, yielding a mixture of the dihydronepetalactones **c** and **d** was essentially the same as described above (Scheme 4).

Transformation of the free hydroxy group to the TBDMS ethers **27**/27* was followed by cleavage of the acetate moiety with methanolic KOH to give a mixture of the alcohols **28**/28*. Oxidation of the primary alcohol function with ruthenium(III) chloride and sodium periodate in a biphasic mixture of carbon tetrachloride, acetonitrile and phosphate buffer (pH 7) afforded the carboxylic acids **29**/29* without epimerization at C1 [37–38]. After cleavage of the TBDMS ether with HF in acetonitrile, a mixture of dihydronepetalactones **c** and **d** was formed by lactonization of the hydroxy acids **18**/18* with DCC and DMAP in dichloromethane at rt. The C4 epimeric dihydronepetalactones **c** and **d** could be separated by column chromatography over silica. Starting from the enantiomer of **26**, a mixture of dihydronepetalactones **c'** and **d'** was synthesized by following the same reaction sequence.

### Formal synthesis of a mixture of *trans*-fused dihydronepetalactones **a** and **b** from (*R*)-limonene

**Scheme 6:**
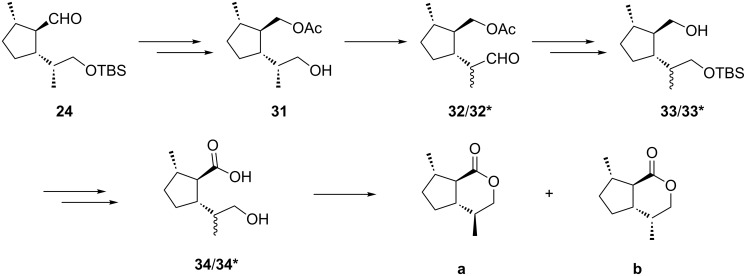
Formal synthesis of a mixture of dihydronepetalactones **a** and **b** from (*R*)-limonene.

As outlined above, six of the eight possible stereoisomers of *trans*-fused dihydronepetalatones were synthesized from the enantiomers of limonene following a new route. Compound **a** and its enantiomer **a'** were prepared according to the procedure described by Wolinsky [20–21]. However, our new approach also includes a formal synthesis of **a** and **a'**. A mixture of **a** and **b** will be easily obtained from the protected hydroxy aldehyde **24** by the straight forward procedure outlined in Scheme 6.

Reduction of the aldehyde function of **24** and acetylation of the resulting primary alcohol followed by cleavage of the silyl group will furnish the primary alcohol **31**, which upon oxidation will yield the corresponding aldehyde that can be epimerized to the diastereomers **32**/32* as shown above. Subsequent reduction of **32**/32*, silylation of the resulting primary alcohols and saponification will produce a mixture of the diastereoisomers **33**/33*. Oxidation of the primary alcohol moiety, followed by cleavage of the silyl group will yield the epimeric hydroxy acids 34/34* which will form a mixture of the dihydronepetalactones **a** and **b** after lactonization. As shown above, this mixture can be separated upon column chromatography.

## Conclusion

In summary, we synthesized all eight *trans*-fused stereoisomeric dihydronepetalactones. After having used the enantiomers of pulegone as educts in Wolinsky’s route to (4*S*,4a*S*,7*S*,7a*R*)-dihydronepetalactone (**a**) and its enantiomer **a'** [23], we developed an improved and general way for the synthesis of all *trans*-fused dihydronepetalactones, starting from pure enantiomers of limonene. Our approach is also superior to that starting from optically active carvone that yields the starting material for the synthesis of *trans*-fused iridoid lactones only as a byproduct [15].

## Identification of a *trans*-fused dihydronepetalactone in the parasitoid wasp *Alloxysta victrix*

Upon gas chromatography using FFAP as a polar achiral stationary phase, the stereoisomers **a** and **c** could be well separated while **b** and **d** coeluted. However, the latter pair could be resolved on a less polar DB5-capillary, where **b**/**b'** eluted after **d**/**d'** (data not shown). As a result, the relative configuration of each of the *trans*-fused dihydronepetalactones could be unambiguously assigned by GC/MS.

With the exception of (4*S*,4a*S*,7*R*,7a*R*)-dihydronepetalactone (**d**) and its enantiomer **d'**, the stereoisomers could well be distinguished by enantioselective gas chromatography using a 1:1-mixture of OV1701 and heptakis-(6-*O*-*tert*-butyldimethylsilyl-2,3-di-*O*-methyl)-β-cyclodextrin as an optically active stationary phase. Figure 3 shows the corresponding retention times of all eight stereoisomers that were obtained with the two used capillary column systems. Coupled GC/MS using FFAP as the stationary phase revealed the target natural iridoid lactone **X** to show the same mass spectrum and the same retention time as **a**/**a'**, the first eluting pair of the synthetic dihydronepetalactones (Figure 3). Enantioselective gas chromatography on a cyclodextrin column showed **X** to coelute with **a'** which was well separated from its enantiomer by an α-value of **a'**:**a** = 1.01 (Figure 3). Consequently, the structure of **X** was unambiguously assigned to be (4*R*,4a*R*,7*R*,7a*S*)-dihydronepetalactone. It should be noted that Meinwald et al. identified **a**/**a'** (absolute configuration not assigned) as a component of secretions of the abdominal defense glands of the rove beetle *Creophilus maxillosus* [39]. Interestingly, the structure of **a'** is relatively close to that of nepetalactone **7** and lactol **8**, the sex pheromone of the grain aphid *S. avenae* [13] which keeps the second level in the investigated tetratrophic system. Grant et al., found the *trans*-fused (1*R*,4a*S*,7*R*,7a*R*)-1-methoxy-4,7-dimethyl-1,4a,5,6,7,7a)-hexahydrocyclopenta[*c*]pyran, called (1*R*)-1-methoxymyodesert-3-ene, among the volatiles of the Ellangowan poison bush, which they transformed to the corresponding lactone **a'** [24]. Apart from this compound and very few others, the stereogenic center carrying the methyl group in the five-membered ring of iridoid lactones including insect semiochemicals [13–15] generally shows (*S*)-configuration. Only recently, two isomeric iridoid lactones showing (7*R*)-configuration have been identified from the *Drosophila* parasitoid *Leptopilina heterotoma* [40]. Compound **X** has been identified in the mandibular gland secretions of other *Alloxysta* species, too, [41]. However, its biological significance is not yet clear and will need further investigations.

The differentiation of the oxygen containing functional groups in the trisubstituted cyclopentene **16**, a key-compound in our synthetic approach, provides access to a large number of iridoids including nepetalactones but also iridomyrmecins and monocyclic compounds. Consequently, having reference compounds at hand, structures of hitherto unknown iridoids [42] may now be assigned. It may turn out that the chiral center carrying the methyl group in the five-membered ring of iridoids may much more often show (*R*)-configuration than it is known today.

## Supporting Information

File 1 Experimental details and characterization data for synthesized compounds.

## References

[1] Hilgraf R, Zimmermann N, Lehmann L, Tröger A, Francke W (2012). Beilstein J Org Chem.

[2] Lilley R, Hardie J, Merritt L A, Pickett J A, Wadhams L J, Woodcock C M (1994). Chemoecology.

[3] Dawson G W, Pickett J A, Smiley D W M (1996). Bioorg Med Chem.

[4] Petersen G, Matthiesen C, Francke W, Wyss U (2000). Eur J Entomol.

[5] Höller C, Micha S G, Schulz S, Francke W, Pickett J A (1994). Experientia.

[6] Petersen G, Matthiesen C, Stolzenberg N, Zimmermann N, Hilgraf R, Lehmann L, Francke W, Wyss U (2001). Mitt Dtsch Entomol Ges.

[7] Regnier F E, Waller G R (1972). Biochemical Application of Mass Spectrometry.

[8] Sakai T, Nakajima K, Sakan T (1980). Bull Chem Soc Jpn.

[9] McElvain S M, Bright R D, Johnson P R (1941). J Am Chem Soc.

[10] Bates R B, Eisenbraun E J, McElvain S M (1958). J Am Chem Soc.

[11] Eisenbraun E J, Browne C E, Irvin-Willis D J, McGurk E L, Eliel E L, Harris D L (1980). J Org Chem.

[12] Cavill G W K, Clark D V (1967). J Insect Physiol.

[13] Pickett J A, Wadhams L J, Woodcock C M, Hardie J (1992). Annu Rev Entomol.

[14] Goldansaz S H, Dewhirst S, Birkett M A, Hooper A M, Smiley D W M, Pickett J A, Wadhams L, McNeil J N (2004). J Chem Ecol.

[15] Hooper A M, Donald B, Woodcock C M, Park J H, Paul R L, Boo K S, Hardie J, Pickett J A (2002). J Chem Ecol.

[16] Ficini J, d’Angelo J (1976). Tetrahedron Lett.

[17] Lee E, Yoon C H (1994). J Chem Soc, Chem Commun.

[18] Nangia A, Prasuna G, Rao P B (1997). Tetrahedron.

[19] Beckett J S, Beckett J D, Hofferberth J E (2010). Org Lett.

[20] Wolinsky J, Gibson T, Chan D, Wolf H (1965). Tetrahedron.

[21] Wolinsky J, Eustace E J (1972). J Org Chem.

[22] Achmad S A, Cavill G W K (1963). Aust J Chem.

[23] Ibarra-Wiltschek D (1995). Identifizierung und Synthese mono- und sesquiterpenoider Inhaltsstoffe aus Hymenopteren.

[24] Grant H G, O’Regan P J, Park R J, Sutherland M D (1980). Aust J Chem.

[25] Auer L, Weymuth C, Scheffold R (1993). Helv Chim Acta.

[26] Naemura K, Hasegawa T, Miyabe H, Chikamatsu H (1992). Bull Chem Soc Jpn.

[27] Wolinsky J, Slabaugh M R, Gibson T (1964). J Org Chem.

[28] Wolinsky J, Nelson D (1969). Tetrahedron.

[29] Lange G L, Neidert E E, Orrom W J, Wallace D J (1978). Can J Chem.

[30] Rao H S P, Reddy K S (1994). Tetrahedron Lett.

[31] Abiko A, Roberts J C, Takemasa T, Masamune S (1986). Tetrahedron Lett.

[32] Crabtree R H, Davis M W (1983). Organometallics.

[33] Crabtree R H, Davis M W (1986). J Org Chem.

[34] Brown J M (1987). Angew Chem, Int Ed Engl.

[35] Ohta T, Miyake T, Seido N, Kumobayashi H, Takaya H (1995). J Org Chem.

[36] Takaya H, Ohta T, Sayo N, Kumobayashi H, Akatagawa S, Inoue S, Kasahara I, Noyori R (1987). J Am Chem Soc.

[37] Carlsen P H J, Katsuki T, Martin V S, Sharpless K B (1981). J Org Chem.

[38] Mori K, Ebata T (1986). Tetrahedron.

[39] Jefson M, Meinwald J, Nowicki S, Hicks K, Eisner T (1983). J Chem Ecol.

[40] Stökl J, Hofferberth J, Pritschet M, Brummer M, Ruther J (2012). J Chem Ecol.

[41] Hübner G, Völkl W, Francke W, Dettner K (2002). Biochem Syst Ecol.

[42] Huth A, Dettner K (1990). J Chem Ecol.

