# Supplementary material for: Stereoselective synthesis of trans-fused iridoid lactones and their identification in the parasitoid wasp Alloxysta victrix, Part I: Dihydronepetalactones
Source: Beilstein J Org Chem. 2012 Aug 7;8:1246–55. doi: 10.3762/bjoc.8.140 (PMC3458745; doi:10.3762/bjoc.8.140)
Supplement: File 1 — Experimental details and characterization data for synthesized compounds. [file Beilstein_J_Org_Chem-08-1246-s001.pdf]

Supporting Information

for

Stereoselective synthesis of *trans*-fused iridoid lactones and their identification in the parasitoid wasp *Alloxysta victrix*, Part I: Dihydronepetalactones

Nicole Zimmermann, Robert Hilgraf, Lutz Lehmann, Daniel Ibarra, and Wittko Francke*

Address: Department of Chemistry - Organic Chemistry, University of Hamburg, Martin-Luther-King-Platz 6, D-20146 Hamburg

Email: Wittko Francke - francke@chemie.uni-hamburg.de

* Corresponding author

Experimental details and characterization data for synthesized compounds

General remarks:

^{1}H and ^{13}C NMR spectra were recorded with a Bruker DRX 500 (^{1}H: 500 MHz, ^{13}C: 126 MHz) or with a Bruker AMX 400 (^{1}H: 400 MHz, ^{13}C: 101 MHz) spectrometer. Chemical shifts were referenced to the corresponding residual solvent signal, i.e., δ_H = 7.26 and δ_C = 77.0 ppm for $CDCl_3$. Coupled gas chromatography/mass spectrometry (GC/MS)-analyses at low resolution were run at 70 eV, EI, using a Fisons GC8008/MD800 equipped with an Optima 5MS column (30 m, 0.25 m id fused silica capillary) 3 min 50 °C, then programmed to 220 °C at a rate of 5 °C/min using helium as the carrier gas. High resolution GC/MS-analyses, GC/HRMS–EI (RP:5000) and GC/CIMS (RP:600) were carried out by using a double focusing mass spectrometer VG 70/250 SE (Vacuum Generators, Manchester, UK) linked to a gas chromatograph HP 5890 (Hewlett Packard, Palo Alto, CA, USA). Separation conditions were the same as those used for low resolution GC/MS: Standard gas chromatography was carried out by using a Satochrom (Fisons instruments) equipped with fused silica capillaries. Hydrogen served as the carrier gas. The following columns were employed: a) FFAP (50 m, 0.25 mm id, Macherey-Nagel, Düren, Germany) 3 min at 50 °C, then programmed to 220 °C at a rate of 5°C/min; b) DB 5 (50 m, 0.25 mm id, J & W Scientitic Folsom CA, US) 5 min at 60 °C, then programmed to 300 °C at a rate of 5°C/min; c) 1:1 mixture of OV1701 and heptakis-(6-*O*-*tert*-butyldimethylsilyl-2,3-di-*O*-methyl)-β-cyclodextrin (25 m, 0.25 mm id, tailor-made at our lab) 5 min at 60 °C , then programmed to 160 °C at a rate of 3 °C/min. Flash chromatography was carried out using Merck silica gel 60 (240–400 mesh). All experiments were carried out in oven-dried glassware under a dry argon atmosphere. Standard vacuum techniques were used for the handling of air-sensitive materials. Solvents were dried and kept under N_2 and freshly distilled before use. Reagents were used as commercially available.

(5*S*)-1-Formyl-2-methyl-5-(1-methylethenyl)-1-cyclopentene (15): A solution of 40 g (293 mmol) of (*R*)-limonene (**14**) and pyridine (6.7 mL) in methanol (240 mL) was cooled to −50 °C, and ozone was bubbled through the solution for 12 h followed by argon for 30 min. Subsequently, 33.5 mL (462 mmol) dimethylsulfide was added slowly. The reaction mixture was stirred overnight at −50 °C, then allowed to warm to rt before water (1 L) was added. The phases were separated, and the aqueous phase was extracted with chloroform (6 x 200 mL). The combined organic solutions were dried over magnesium sulfate and concentrated in vacuo. The residue was dissolved in 350 mL diethyl ether and washed with 35 mL 5% hydrochloric acid, 35 mL saturated aqueous solutions of sodium hydrogen carbonate and brine (3 x 35 mL), and dried over magnesium sulfate. The organic solutions were concentrated in vacuo and the residue was purified by chromatography over silica (hexane/ethyl acetate 1:1) to give 42.5 g (86%) of (3*R*)-3-(1-methylethenyl)-6-oxoheptanal as a colorless oil. ^{1}H NMR (400 MHz, $CDCl_3$): δ = 1.62 (m, 2H, 1'-H), 1.64 (s, 3H, 4-CH_3), 2.14 (s, 3H, 4'-H), 2.40 (t, $^3J_{H,H}$ = 7.1 Hz, 2H, 2'-H), 2.47 (m, 2H, 2-H), 2.68 (m, 1H, 3-H), 4.81 (m, 2H, 5-H), 9.68 (t, $^3J_{H,H}$ = 2.4 Hz, 1H, CHO); ^{13}C NMR (101 MHz, $CDCl_3$): δ = 18.0, 26.0, 29.6, 40.3, 40.5, 47.0, 112.8, 144.6, 201.3, 207.8; MS *m*/*z* (%): 168 (0) $[M]^+$, 107 (18), 82 (11), 71 (11), 69 (12), 67 (22), 58 (13), 55 (18), 53 (10), 43 (100), 41 (37), 40 (15), 39 (23).

A solution of 34.8 g (3*R*)-3-(1-methylethenyl)-6-oxoheptanal (207 mmol), piperidine (2.5 mL) and glacial acetic acid (2.5 mL) in benzene (250 mL) was heated under reflux for 45 min using a Dean-Stark-trap. After cooling to rt, the organic phase was washed with 5% hydrochloric acid (2 x 75 mL), with saturated aqueous hydrogen carbonate (2 x 70 mL) and with brine (3 x 70 mL). After drying over magnesium sulfate, the organic layer was concentrated in vacuo to give 26.4 g (85%) of the aldehyde **15** as a yellow oil. ^{1}H NMR (400 MHz, $CDCl_3$): δ = 1.68 (s, 3H, $CH_3C{=}CH_2$),

2.10 (m, 2H, 3-H), 2.18 (s, 3H, 2-CH_3), 2.46 (m, 1H, 4-H_a), 2.63 (m, 1H, 4-H_b), 3.62 (m, 1H, 5-H), 4.61 (s, 1H, $CH_3C{=}CH_aH_b$), 4.61 (s, 1H, $CH_3C{=}CH_aH_b$), 10.00 (s, 1H, CHO); ^{13}C NMR (101 MHz, $CDCl_3$): δ = 14.5, 20.5, 28.6, 39.5, 50.8, 109.8, 139.2, 146.9, 163.5, 188.0; MS *m/z* (%): 151 (4) $[M + H]^+$, 150 (29) $[M]^+$, 135 (28), 122 (21), 108 (12), 107 (73), 105 (20), 93 (26), 91 (64), 81 (34), 79 (100), 77 (51), 53 (26), 51 (21), 41 (45), 39 (68).

Anal. calcd for $C_{10}H_{14}O$: C, 79.96; H, 9.39; found C, 80.0; H, 9.32%.

(5*R*)-1-Formyl-2-methyl-5-(1-methylenthenyl)-1-cyclopentene (15'): Ozonolysis of 40.0 g (293 mmol) of (*S*)-limonene (**14'**), following the procedure as described above, yielded 44.9 g (91%) of (3*S*)-3-(1-methylethenyl)-6-oxoheptanal as an oil. The NMR and mass spectra were identical to those of the (3*R*)-product. Intramolecular aldol condensation of 72.3 g (430 mmol) of (3*S*)-3-(1-methylethenyl)-6-oxoheptanal as described for the synthesis of **15** yielded 50.8 g (79%) of **15'**. The NMR and mass spectra were identical to those of **15**.

(5*S*)-1-Hydroxymethyl-2-methyl-5-(1-methylethenyl)-1-cyclopentene (19): To a suspension of 4.45 g (117 mmol) of $LiAlH_4$ in diethyl ether (190 mL) was added dropwise a solution of 25.0 g (166 mmol) of the aldehyde **15** in diethyl ether (290 mL) at rt. The reaction mixture was continued to stir at rt for 1 h and then quenched by careful addition of 15 mL saturated aqueous ammonium chloride. The resulting precipitate was filtered off and washed with 1 L diethyl ether. The organic solution was dried over magnesium sulfate, and the solvent was removed in vacuo to give 20.7 g (82%) of the alcohol **19** as a pale yellow oil. ^1H NMR (400 MHz, $CDCl_3$): δ = 1.64 (s, 3H, $CH_3C{=}CH_2$), 1.69 (m, 1H, 4-H_a), 1.74 (s, 3H, 2-CH_3), 2.05 (m, 1H, 4-H_b), 2.28 (m, 1H, 3-H_a), 2.39 (m, 1H, 3-H_b), 3.47 (m, 1H, 5-H), 4.01 (d, $^3J_{H,H}$ = 12.2 Hz,

1H, 1-CH_a), 4.21 (d, $^3J_{H,H}$ = 12.2 Hz, 1H, 1-CH_b), 4.76 (m, 2H, $CH_3C{=}CH_2$); ^{13}C NMR (101 MHz, $CDCl_3$): δ = 13.6, 18.4, 27.3, 37.4, 54.3, 57.6, 110.2, 134.9, 137.9, 148.5; MS *m*/*z* (%): 152 (9) $[M]^+$, 119 (96), 109 (33), 93 (66), 91 (99), 81 (56), 79 (64), 77 (64), 67 (45), 55 (48), 53 (40), 43 (44), 41(100), 39 (99); Anal. calcd for $C_{10}H_{16}O$: C, 78,90; H, 10.59; found: C, 78.9; H, 10.55%.

(5*R*)-1-Hydroxymethyl-2-methyl-5-(1-methylethenyl)-1-cyclopentene (19'): Applying the same procedure to 50.7 g (338 mmol) of **15'** yielded 41.1 g (80%) of **19'**. The NMR and mass spectra were identical to those of **19**.

(5*S*)-1-Acetoxymethyl-2-methyl-5-(1-methylethenyl)-1-cyclopentene (20): A mixture of 79.25 g (520 mmol) of the alcohol **19** in acetic anhydride (860 mL) and pyridine (12.3 mL) was stirred at rt overnight. The reaction mixture was poured into diethyl ether (2 L) and washed with water (6 x 400 mL). The organic phase was dried over magnesium sulfate and concentrated in vacuo to give 92.4 g (91%) of the acetate **20** as a pale yellow oil. ^{1}H NMR (400 MHz, $CDCl_3$): δ = 1.60 (s, 3H, 2-CH_3), 1.68 (m, 2H, 3-H), 1.76 (s, 3H, $CH_3C{=}CH_2$), 2.03 (s, 3H, acetyl-CH_3), 2.30 (m, 1H, 4-H_a), 2.39 (m, 1H, 4-H_b), 3.43 (m, 1H, 5-H), 4.38 (d, $^3J_{H,H}$ = 11.2 Hz, 1H, 1-CH_a), 4.69 (m, $^3J_{H,H}$ = 12.2 Hz, 3H, 1-CH_b and $CH_3C{=}CH_2$); ^{13}C NMR (101 MHz, $CDCl_3$): δ = 14.2, 18.8, 20.9, 27.8, 37.8, 54.9, 59.5, 110.8, 131.0, 141.4, 147.6, 171.2; MS *m*/*z* (%): 194 (0) $[M]^+$, 134 (42), 119 (100), 105 (22), 93 (30) 91 (64), 79 (25), 77 (29), 41 (45), 39 (32); Anal. calcd for $C_{12}H_{18}O_2$: C, 74.19; H, 9.34; found: C, 74.1; H, 9.3%.

(5*R*)-1-Acetoxymethyl-2-methyl-5-(1-methylethenyl)-1-cyclopentene (20'): Applying the same procedure to 82.8 g (540 mmol) of **19'** yielded 101.2 g (96%) of **20'**. The NMR and mass spectra were identical to those of **20**.

(5*S*)-1-Acetoxymethyl-2-methyl-5-(2-hydroxy-(1*R*)-methylethyl)-1-cyclopentene (16): To a stirred solution of 125 mL (1.16 mol) of 2-methyl-2-butene in 320 mL of tetrahydrofuran at −18 °C was added dropwise 265 mL of a 1 M solution of borane in THF. Stirring of this mixture was continued for 2.5 h at this temperature. After warming to 0 °C, a solution of 36.85 g (190 mmol) of the acetate **20** in tetrahydrofuran (100 mL) was added dropwise. The reaction mixture was stirred for 1 h at 0 °C and then at rt overnight. After cooling to −18 °C, water (5.8 mL) was added dropwise, followed by 3 M sodium hydroxide (160 mL) and 160 mL of 30% aqueous hydrogen peroxide (160 mL). Stirring was continued for 1 h at 0 °C and then at rt overnight. The reaction mixture was washed with 500 mL of a 25% aqueous solution of potassium hydroxide, and the aequous phase was extracted with diethyl ether (3 x 300 mL). The combined organic solutions were washed with brine (250 mL), dried over magnesium sulfate, and concentrated in vacuo. The residue was purified by chromatography over silica (hexane/ethyl acetate 4:1, then 1:1) to give the alcohol **16** (32.5 g, 66%) as a pale yellow oil. ^{1}H NMR (400 MHz, $CDCl_3$): δ = 0.97 (d, $^{3}J_{H,H}$ = 7.1 Hz, 3H, 1'-CH_3), 1.64 (m, 2H), 1.74 (s, 3H, 2-CH_3), 1.89 (m, 2H), 2.04 (s, 3H, acetyl-CH_3), 2.29 (m, 2H), 2.86 (br s, 1H, OH), 3.35 (dd, $^{3}J_{H,H}$ = 7.9, 10.7 Hz, 1H, 2'-H_a), 3.56 (dd, $^{3}J_{H,H}$ = 5.6, 10.7 Hz, 1H, 2'-H_b), 4.64 (d, $^{3}J_{H,H}$ = 20.4 Hz, 1H, 1-CH_a), 4.68 (d, $^{3}J_{H,H}$ = 20.4 Hz, 1H, 1-CH_b); ^{13}C NMR (101 MHz, $CDCl_3$): δ = 14.2, 15.9, 21.0, 23.7, 37.7, 37.8, 50.2, 59.8, 65.3, 131.2, 141.5, 171.3; MS *m*/*z* (%): 212 (0) $[M]^+$, 137 (37), 121 (41), 119 (46), 95 (45), 93 (100), 91 (80), 79 (57), 77 (46), 43 (65), 41 (38), 39 (52); Anal. calcd for $C_{12}H_{20}O_3$: C, 67.89; H, 9.50; found: C, 67.8, H, 9.47%.

(5*R*)-1-Acetoxymethyl-2-methyl-5-(2-hydroxy-(1*S*)-methylethyl)-1-cyclopentene (16’):

Applying the same procedure to 37.4 g (190 mmol) of **20’** yielded 28.2 g (69%) of **16’**. The NMR and mass spectra were identical to those of **16**.

(5*S*)-1-Acetoxymethyl-5-(2-*tert*-butyldimethylsilyloxy-(1*R*)-methylethyl)-2-methyl-1-cyclopentene (21): To a solution of 55.0 g (260 mmol) of the acetate **16** and 43.12 g (630 mmol) of imidazole in DMF (650 mL) was added 48.2 g (320 mmol) of TBDMSCl in small portions at −10 °C. The reaction mixture was stirred for 3 h at 0 °C. Subsequently, saturated aqueous sodium hydrogen carbonate (1 L) was added, and the aqueous phase was extracted with hexane (3 x 600 mL). The combined organic solutions were washed with water (3 x 900 mL) and brine (2 x 900 mL), dried over magnesium sulfate, and concentrated in vacuo to give 83.8 g (99%) of the TBDMS ether **21** as a pale yellow oil. ^{1}H NMR (400 MHz, $CDCl_3$): δ = −0.02 (s, 6H, $Si(CH_3)_2$, 0.86 (s, 9H, $C(CH_3)_3$), 0.94 (d, $^{3}J_{H,H}$ = 7.1 Hz, 3H, 1’-CH_3), 1.63 (m, 2H, 4H), 1.72 (s, 3H, 2-CH_3), 1.87 (m, 1H), 1.95 (m, 1H), 2.05 (m, 3H, acetyl-CH_3), 2.25 (m, 2H, 4-H), 3.31 (dd, $^{3}J_{H,H}$ = 7.9, 9.7 Hz, 1H, 2’-H_a), 3.47 (dd, $^{3}J_{H,H}$ = 5.1, 9.7 Hz, 1H, 2’-H_b), 4.57 (d, $^{3}J_{H,H}$ = 12.2 Hz, 1H, 1-CH_a), 4.69 (d, $^{3}J_{H,H}$ = 12.2 Hz, 1H, 1-CH_b); ^{13}C NMR (101 MHz, $CDCl_3$): δ = −3.2, 14.6, 16.3, 21.4, 24.3, 26.3, 32.0, 37.9, 38.0, 50.5, 60.3, 65.4, 131.8, 140.9, 171.6; MS *m*/*z* (%): 326 (0) $[M]^+$, 135 (100), 134 (34), 121 (20), 119 (34), 117 (37), 107 (32), 95 (39), 93 (46), 91 (20), 77 (22), 75 (56), 73 (34), 59 (24), 43 (57), 41 (23); Anal. calcd for $C_{18}H_{34}O_3Si$: C, 66.21; H, 10.49; found: C, 66.1; H, 10.43%.

(5*R*)-1-Acetoxymethyl-5-(2-*tert*-butyldimethylsilyloxy-(1*S*)-methylethyl)-2-methyl-1-cyclopentene (21'): Applying the same procedure to 50.0 g (236 mmol) of **16'** yielded 76.2 g (99%) of **21'**. The NMR and mass spectra were identical to those of **21**.

(5*S*)-1-Hydroxymethyl-5-(2-*tert*-butyldimethylsilyloxy-(1*R*)-methylethyl)-2-methyl-1-cyclopentene (22): To a solution of 43.3 g (133 mmol) of the acetate **21** in methanol (140 mL) was added dropwise 800 mL of a 2 M solution of potassium hydroxide in methanol at 0 °C. After stirring at rt for 2 h, the reaction mixture was poured into 500 mL ice water and extracted with diethyl ether (4 x 500 mL). The combined organic layers were washed with brine (400 mL), dried over magnesium sulfate and concentrated in vacuo to give 32.0 g (87%) of the alcohol **22** as a pale yellow oil. ^{1}H NMR (400 MHz, $CDCl_3$): δ = −0.01 (s, 6H, $Si(CH_3)_2$, 0.80 (d, $^3J_{H,H}$ = 7.1 Hz, 3H, 1'-CH_3), 0.83 (s, 9H, $C(CH_3)_3$), 1.56 (m, 2H), 1.64 (s, 3H, 2-CH_3), 1.84 (m, 1H), 2.24 (m, 3H), 2.90 (br s, 1H, OH), 3.46 (m, 2H, 2'-H), 4.08 (d, $^3J_{H,H}$ = 12.0 Hz, 1H, 1-CH_a), 4.17 (d, $^3J_{H,H}$ = 12.0 Hz, 1H, 1-CH_b); ^{13}C NMR(101 MHz, $CDCl_3$): δ = −3.6, 14.1, 15.7, 23.9, 26.0, 32.0, 37.8, 37.9, 50.0, 58.6, 66.1, 136.1, 136.8; MS *m*/*z* (%): 284 (0) $[M]^+$, 135 (60), 121(37), 119 (42), 115 (29), 107 (33), 105 (21), 95 (68), 93 (54), 91 (29), 79 (22), 77 (26), 75 (100), 73 (43), 59 (23), 55 (21), 41 (25); Anal. calcd for $C_{16}H_{32}O_2Si$: C, 67.54; H, 11.34; found: C, 67.6; H, 11.30%.

(5*R*)-1-Hydroxymethyl-5-(2-*tert*-butyldimethylsilyloxy-(1*S*)-methylethyl)-2-methyl-1-cyclopentene (22'): Applying the same procedure to 39.4 g (121 mmol) of **21'** yielded 29.9 g (87%) of **22'**. The NMR and mass spectra were identical to those of **22**.

(5*S*)-1-Formyl-5-(2-*tert*-butyldimethylsilyloxy-(1*R*)-methylethyl)-2-methyl-1-cyclopentene (23): To a solution of 31.9 g (112 mmol) of the alcohol **22** in dichloromethane (840 mL) were added 20 g of 4 Å grinded molecular sieves. The suspension was stirred at rt for 15 min, cooled to 0 °C, and 78 g (207 mmol) of pyridinium dichromate was added in small portions. The reaction mixture was stirred for 1 h at 0 °C followed by 2 h at rt. The suspension was filtered through a short silica column and washed with diethyl ether (4 x 400 mL). The combined organic solutions were concentrated in vacuo, and the residue was purified by chromatography over silica (hexane/ethyl acetate 4:1) to give 19.2 g (61%) of the aldehyde **23** as a pale yellow oil. ^{1}H NMR (400 MHz, $CDCl_3$): δ = −0.02 (s, 6H, $Si(CH_3)_2$), 0.86 (s, 9H, $C(CH_3)_3$), 0.90 (d, $^{3}J_{H,H}$ = 6.6 Hz, 3H, 1'-CH_3), 1.26 (m, 1H), 1.85 (m, 2H), 2.13 (s, 3H, 2-CH_3), 2.39 (m, 1H), 2.56 (m, 1H), 3.33 (dd, $^{3}J_{H,H}$ = 6.6, 9.7 Hz, 1H, 2'-H_a), 3.49 (dd, $^{3}J_{H,H}$ = 5.1, 9.7 Hz, 1H, 2'-H_b), 10.00 (s, 1H, CHO); ^{13}C NMR(100.6 MHz, $CDCl_3$): δ = −3.6, 14.6, 15.7, 24.4, 26.0, 32.0, 37.4, 39.7, 47.2, 65.7, 139.9, 163.4, 188.5; MS *m*/*z* (%): 282 (0) $[M]^+$, 225 (29), 167 (26), 93 (32), 81 (23), 75 (100), 73 (33), 59 (37), 41 (29); Anal. calcd for $C_{16}H_{30}O_2Si$: C, 68.03; H, 10.70; found: C, 68.1; H 10.65%.

(5*R*)-1-Formyl-5-(2-*tert*-butyldimethylsilyloxy-(1*S*)-methylethyl)-2-methyl-1-cyclopentene (23'): Applying the same procedure to 29.8 g (105 mmol) of **22'** yielded 20.1 g (68%) of **23'**. The NMR and mass spectra were identical to those of **23**.

(1*R*,2*S*,5*S*)-1-Formyl-2-(2-*tert*-butyldimethylsilyloxy-(1*R*)-methylethyl)-5-methylcyclopentane (24): To a solution of 7.5 g (26.5 mmol) of the aldehyde **23** in methanol (500 mL) was added 8.4 g (133.2 mmol) of ammonium formate followed by 375 mg 10% Pd/C, and the resulting mixture was heated under reflux for 5 h. After

cooling to rt the reaction mixture was filtered over Celite and washed with diethyl ether (750 mL). The organic solution was washed with 2 M hydrochloric acid (2 x 80 mL) and brine (50 mL), dried over magnesium sulfate, and concentrated in vacuo. The residue was purified by chromatography over silica (hexane/ethyl acetate 20:1) to give 3.6 g (48%) of the aldehyde **24** as a pale yellow oil. ^{1}H NMR (500 MHz, $CDCl_3$): δ = −0.01 (s, 6H, $Si(CH_3)_2$), 0.85 (d, $^3J_{H,H}$ = 6.9 Hz, 3H, 1'-CH_3), 0.88 (s, 9H, $C(CH_3)_3$), 1.01 (d, $^3J_{H,H}$ = 6.3 Hz, 3H, 5-CH_3), 1.31 (m, 1H, 4-H_a), 1.55 (m, 1H, 3-H_a), 1.63 (m, 1H, 1'-H), 1.84 (m, 2H, 3-H_b and 4-H_b), 2.07 (m, 1H, 1-H), 2.13 (m, 1H, 5-H), 2.31 (m, 1H, 2-H), 3.39 (dd, $^3J_{H,H}$ = 6.3, 10.1 Hz, 1H, 2'-H_a), 3.48 (dd, $^3J_{H,H}$ = 5.5, 10.1 Hz, 1H, 2'-H_b), 9.50 (d, $^3J_{H,H}$ = 7.0 Hz, 1H, CHO); ^{13}C NMR(126 MHz, $CDCl_3$): δ = −0.0, 15.1, 18.3, 19.1, 25.9, 26.4, 27.9, 28.2, 35.3, 43.6, 63.4, 66.7, 204.7; MS *m*/*z* (%): 284 (0) $[M]^+$, 135 (39), 121 (24), 115 (25), 107 (29), 93 (44), 81 (25), 75 (100), 73 (42), 59 (27), 55 (26), 43 (22), 41 (32); Anal. calcd for $C_{16}H_{32}O_2Si$: C, 67.54; H, 11.34; found: C, 67.6; H, 11.38%.

(1*S*,2*R*,5*R*)-1-Formyl-2-(2-*tert*-butyldimethylsilyloxy-(1*S*)-methylethyl)-5-methylcyclopentane (24'): Applying the same procedure to 10.0 g (35.4 mmol) of **23'** yielded 3.8 g (38%) of **24'**. The NMR and mass spectra were identical to those of **24**.

(1*R*,2*S*,5*S*)-1-Carboxy-2-(2-*tert*-butyldimethylsilyloxy-(1*R*)-methylethyl)-5-methylcyclopentane (25): A solution of 4.72 g (16.6 mmol) of the aldehyde **24** was dissolved in *tert*-butanol, and the pH of the reaction solution was adjusted to pH 4.5 by the addition of 60 mL of a 5% aqueous solution of sodium dihydrogen phosphate. Subsequently, 99 mL of a 1 M aqueous solution of potassium permanganate was added dropwise at 0 °C, and the resulting mixture was vigorously stirred at rt for

2.5 h. A saturated aqueous solution of $NaHSO_3$ was added until the reaction mixture became colorless. After adjustment to pH 3 with 0.5 M HCl and extraction with ether (4 x 300 mL), the combined organic extracts were dried over $MgSO_4$ and concentrated in vacuo. Chromatography over silica (hexane:ethyl acetate 1:1) afforded 1.1 g (22%) of the carboxylic acid **25** as a pale yellow oil. ^{1}H NMR (400 MHz, $CDCl_3$): δ = −0.01 (s, 6H, $Si(CH_3)_2$), 0.84 (s, 9H, $C(CH_3)_3$), 0.86 (d, $^3J_{H,H}$ = 7.1 Hz, 3H, 1'-CH_3), 1.01 (d, $^3J_{H,H}$ = 6.3 Hz, 3H, 5-CH_3), 1.19 (m, 1H,), 1.43 (m, 1H), 1.65 (m, 1H), 1.82 (m, 2H), 2.18 (m, 3H), 3.36 (dd, $^3J_{H,H}$ = 6.4, 10.1 Hz, 1H, 2'-H_a), 3.55 (dd, $^3J_{H,H}$ = 5.6, 9.7 Hz, 1H, 2'-H_b) ppm; ^{13}C NMR (101 MHz, $CDCl_3$): δ = −3.6, 15.1, 19.5, 25.9, 28.7, 32.0, 33.7, 39.8, 40.4, 46.8, 55.8, 66.8, 181.3 ppm; MS *m*/*z* (%): 300 (0) [M^+], 243 (1), 225 (3), 124 (4), 81 (38), 75 (100), 73 (33), 57 (29), 41 (38); Anal. calcd for $C_{16}H_{30}O_3Si$: C, 68.38; H, 10.13; found: C, 64.5; H, 10.10%.

(1*S*,2*R*,5*R*)-1-Carboxy-2-(2-*tert*-butyldimethylsilyloxy-(1*S*)-methylethyl)-5-methylcyclopentane (25'): Applying the same procedure to 6.42 g (22.6 mmol) of **24'** yielded 6.2 g (91%) of **25'**. The NMR and mass spectra were identical to those of **25**.

(1*R*,2*S*,5*S*)-1-Carboxy-2-(2-hydroxy-(1*R*)-methylethyl)-5-methylcyclopentane (17): To a solution of 600 mg (2.0 mmol) of the carboxylic acid **25** in THF (2 mL) was added 4.0 mL of a 1 M (4.0 mmol) solution of tetrabutylammonium fluoride in THF. After stirring for 4.5 h at rt, ether (10 mL) was added, the phases were separated, and the aqueous layer was extracted with ether (3 x 10 mL). The combined organic extracts were dried over $MgSO_4$ and concentrated in vacuo to give 372 mg (100%) of the hydroxy acid **17** as a pale yellow oil which was used without further purification in the next reaction. MS *m*/*z* (%): 186 (0) [M^+], 156 (5), 138 (8), 127 (14), 108 (26), 96

(38), 87 (39), 81 (63), 79 (32), 69 (36), 67 (69), 55 (62), 53 (27), 41 (100), 39 (69); Anal. calcd for $C_{10}H_{18}O_3$: C, 64.49; H, 9.74; found: C, 64.4; H, 9.70%.

(1*S*,2*R*,5*R*)-Carboxy-2-(2-hydroxy-*(*1*R*)-methylethyl)-5-methylcyclopentane (17'): Applying the same procedure to 2.4 g (8.0 mmol) of **25'** yielded 1.5 g (100%) of **17'** as a pale yellow oil. The mass spectrum was identical to that of **17**.

(4*R*,4a*S*,7*S*,7a*R*)-4,7-Dimethylhexahydrocyclopenta[*c*]pyran-1(3*H*)-one (dihydronepetalactone b): To a solution of 372 mg (2.00 mmol) of the hydroxy acid **17** dissolved in CH_2Cl_2 (155 mL) were added 453 mg (2.20 mmol) of DCC and 33 mg (0.27 mmol) of DMAP. After stirring for 1 h at rt, the solvent was removed. The residue was suspended in hexane (50 mL) and filtered, and the filter cake was washed with hexane (150 mL). The combined organic solutions were dried over $MgSO_4$ and concentrated in vacuo. Chromatography over silica (hexane/ethylacetate 5:1) afforded 1.9 g (66%) of dihydronepetalactone **b** as a yellow oil. ^{1}H NMR (500 MHz, $CDCl_3$): δ = 0.93 (d, $^3J_{H,H}$ = 7.1 Hz, 3H, 4-CH_3), 1.17 (d, $^3J_{H,H}$ = 6.6 Hz, 3H, 7-CH_3), 1.34 (m, 1H, 6-H_a), 1.53 (m, 1H, 5-H_a), 1.69 (m, 1H, 5-H_b), 1.92 (dd, $^3J_{H,H}$ = 9.7, 13.7 Hz 1H, 7a-H), 2.00 (m, 1H, 6-H_b), 2.09 (m, 1H, 4a-H), 2.23 (m, 1H, 7-H), 2.36 (m, 1H, 4-H), 3.78 (dd, $^3J_{H,H}$ = 11.4, 11.7 Hz, 1H, 3-H_a), 4.38 (dd, $^3J_{H,H}$ = 6.6, 11.7 Hz, 1H, 3-H_b) ppm; ^{13}C NMR (126 MHz, $CDCl_3$): δ = 12.3 (4-CH_3), 20.6 (7-CH_3), 25.1 (C-5), 28.0 (C-4), 31.9 (C-6), 32.9 (C-7), 43.1 (C-4a), 49.3 (C-7a), 73.9 (C-3), 175.5 (C=O) ppm; MS *m*/*z* (%): 168 (1) [M^+], 138 (6), 126 (6), 113 (29), 110 (32), 95 (51), 82 (29), 81 (100), 69 (40), 68 (28), 67 (64), 55 (31), 53 (29), 41 (82), 39 (71); Anal. calcd for $C_{10}H_{16}O_2$: C, 71.39; H, 9.59; found: C, 71.3; H, 9.55%.

(4*S*,4a*R*,7*R*,7a*S*)-4,7-Dimethylhexahydrocyclopenta[*c*]pyran-1(3*H*)-one (dihydronepetalactone b'): Applying the same procedure to 1.5 g (8.0 mmol) of **17'** yielded 604 mg (45%) of dihydronepetalactone **b'** as a pale yellow oil. The NMR and mass spectra were identical to those of dihydronepetalactone **b**.

(1*R*,2S,5*R*)-1-Acetoxymethyl-2-[2-hydroxy-(1*R*)-methylethyl]-5-methylcyclopentane (26): A solution of 11.0 g (51.8 mmol) of the acetate **16** in 300 mL of dichloromethane was degassed using three freeze-thaw cycles. Subsequently, 4.60 g (5.72 mmol, 11 mol %) of Crabtree's catalyst [Ir(cod)PCy_3(py)]PF_6 was added, and the reaction mixture was hydrogenated for 1 h under a pressure of 1 bar. The solvent was removed in vacuo, and the residue was suspended in diethyl ether (200 mL). The precipitate was filtered off and washed with diethyl ether (1 L). The filtrate was concentrated in vacuo, and the residue was purified by chromatography over silica (hexane/ethyl acetate 4:1, then 1:1) to give 9.0 g (81%) of **26** as a pale yellow oil. ^{1}H NMR (500 MHz, C_6D_6): δ = 0.85 (d, $^{3}J_{H,H}$ = 5.4 Hz, 3H, 1'-CH_3), 0.87 (d, $^{3}J_{H,H}$ = 5.4 Hz, 3H, 5-CH_3), 1.10 (m, 2H, 3-H_a and 4-H_a), 1.42 (m, 1H, 1'-H), 1.50 (m, 1H, 4-H_b), 1.60 (m, 2H, 3-H_b and 2-H), 1.70 (s, 3H, acetyl-CH_3), 1.81 (m, 1H, 5-H), 1.88 (m, 1H, 1-H), 3.20 (dd, $^{3}J_{H,H}$ = 6.6, 10.7 Hz, 1H, 2'-H_a), 3.37 (dd, $^{3}J_{H,H}$ = 5.4, 10.7 Hz, 1H, 2'-H_b), 3.98 (dd, $^{3}J_{H,H}$ = 6.2, 11.2 Hz, 1H, 1-CH_a), 4.01 (dd, $^{3}J_{H,H}$ = 7.3, 11.2 Hz, 1H, 1-CH_b); ^{13}C NMR (126 MHz, $CDCl_3$): δ = 13.4, 13.8, 19.3, 28.0, 32.7, 35.6, 38.8, 41.3, 42.4, 64.5, 65.0, 168.9; MS *m*/*z* (%): 214 (0) $[M]^+$, 123 (30), 95 (88), 81 (46), 79 (23), 67 (30), 55 (27), 43 (100), 41 (35); Anal. calcd for $C_{12}H_{22}O_3$: C, 67.26; H, 10.35; found: C, 67.2; H, 10.31%.

(1*S*,2R,5*S*)-1-Acetoxy-2-[2-hydroxy-(1*S*)-methylethyl]-5-methylcyclopentane (26'): Applying the same procedure to 12.5 g (58.9 mmol) of **16'** yielded 10.5 g (83%) of **26'** as an oil. The NMR and mass spectra were identical to those of **26**.

(1*R*,2*R*,5*R*)-1-Acetoxy-2-(2-*tert*-butyldimethylsilyloxy-(1*R*)-methylethyl)-5-methylyclopentane (27): To a solution of 11.7 g (54.7 mmol) of the acetate **26** and 9.3 g (136.6 mmol) of imidazole in DMF (140 mL) at −10 °C was added 9.64 g (66.0 mmol) TBDMSCl in portions over 10 min. After stirring for 3 h at 0 °C, the reaction mixture was poured into 530 mL of saturated aqueous $NaHCO_3$ and extracted with hexanes (4 x 130 mL). The combined organic extracts were washed with 100 mL of water and brine (2 x 100 mL), dried over $MgSO_4$, and concentrated in vacuo to give 17.9 g (100%) of the TBDMS ether **27** as a colorless oil. ^1H NMR (400 MHz, $CDCl_3$): δ = −0.01 (s, 6H, $Si(CH_3)_2$), 0.83 (s, 9H, $C(CH_3)_3$), 0.89 (m, 6H, 5-CH_3 and 1'-CH_3), 1.23 (m, 4H), 1.56 (m, 2H), 1.66 (m, 1H), 1.76 (m, 1H), 2.01 (s, 3H, acetyl-CH_3), 3.34 (dd, $^3J_{H,H}$ = 7.1, 9.7 Hz, 1H, 2'-H_a), 3.55 (dd, $^3J_{H,H}$ = 4.8, 9.7 Hz, 1H, 2'-H_b), 3.96 (m, 2H, 1-CH_2-) ppm; ^{13}C NMR (101 MHz, $CDCl_3$): δ = -5.0, 15.4, 15.8, 21.5, 26.3, 29.5, 32.0, 34.3, 37.0, 40.6, 43.3, 44.4, 66.3, 67.0, 171.7 ppm; MS *m*/*z* (%): 328 (0) [M^+], 211 (2), 137 (78), 117 (97), 95 (97), 81 (100), 75 (46), 73 (30), 57 (20), 43 (44); Anal. calcd for $C_{18}H_{36}O_3Si$: C, 65.80; H, 11.04; found: C, 65.7; H, 11.00%.

(1*S*,2*S*,5*S*)-1-Acetoxy-2-(2-*tert*-butyldimethylsilyloxy-(1*S*)-methylethyl)-5-methylyclopentane (27'): Applying the same procedure to 12.1 g (56.4 mmol) of **26'** yielded 18.5 g (100%) of **27'**. The NMR and mass spectra were identical to those of **27**.

(1*R*,2*R*,5*R*)-1-Hydroxymethyl-2-(2-*tert*-butyldimethylsilyloxy-(1*R*)-methylethyl)-5-methylcyclopentane (28): A solution of 17.9 g (54.7 mmol) of the acetate **27** in MeOH (60 mL) was added dropwise to 330 mL (0.66 mol) of a 2 M solution of KOH in methanol at 0 °C. After stirring for 2.5 h at rt, the reaction mixture was poured into ice-water (400 mL), and extracted with ether (4 x 400 mL). The combined organic extracts were washed with brine (2 x 350 mL), dried over $MgSO_4$, and concentrated in vacuo to give 14.5 g (93%) of the alcohol **28** as a pale yellow oil. ^1H NMR (400 MHz, $CDCl_3$): δ = −0.01 (s, 6H, $Si(CH_3)_2$), 0.83 (d, $^3J_{H,H}$ = 7.1 Hz, 3H, 1'-CH_3), 0.89 (s, 9H, $C(CH_3)_3$), 0.96 (d, $^3J_{H,H}$ = 6.6 Hz, 3H, 5-CH_3), 1.23 (m, 2H), 1.37 (m, 2H), 1.68 (m, 2H), 1.78 (m, 1H), 1.91 (m, 1H), 2.00 (m, 1H), 3.43 (dd, $^3J_{H,H}$ = 8.1, 10.2 Hz, 1H, 2'-H_a), 3.51 (m, 2H, 1-CH_2-), 3.64 (dd, $^3J_{H,H}$ = 5.6, 10.2 Hz, 1H, 2'-H_b) ppm; ^{13}C NMR (101 MHz, $CDCl_3$): δ = −3.6, 13.2, 15.1, 26.1, 29.4, 32.0, 33.9, 37.5, 38.7, 43.1, 44.4, 64.3, 67.0 ppm; MS *m*/*z* (%): 287 (0) [M^+], 199 (5), 137 (65), 105 (75), 81(100), 75 (87), 73 (67), 69 (40), 67 (54), 57 (48), 55 (68), 41 (53); Anal. calcd for $C_{16}H_{34}O_2Si$: C, 67.07; H, 11.96; found: C, 67.0; H, 11.92%.

(1*S*,2*S*,5*S*)-1-Hydroxymethyl-2-(2-*tert*-butyldimethylsilyloxy-(1*S*)-methylethyl)-5-methylcyclopentane (28'): Applying the same procedure to 18.5 g (56.4 mmol) of **27'** yielded 15.7 g (97%) of **28'**. The NMR and mass spectra were identical to those of **28**.

(1*R*,2*S*,5*R*)-1-Carboxy-2-(2-*tert*-butyldimethylsilyloxy-(1*R*)-methylethyl)-5-methylyclopentane (29): In a two phase mixture of CCl_4 (106 mL), acetonitrile (106 mL) and phosphate buffer (159 mL, pH 7) was dissolved 14.5 g (50.7 mmol) of the alcohol **28**. At rt, 32.6 g (151.7 mmol) sodium periodate was slowly added, and stirring was continued for 15 min. After the addition of 250 mg (0.96 mmol) of

ruthenium(III) chloride trihydrate, the resulting mixture was vigorously stirred at rt for 2 h. Subsequently, CH_2Cl_2 (350 mL) and water (350 mL) were added, the layers were separated, and the aqueous phase was extracted with ether (3 x 250 mL). The combined organic extracts were washed with brine (300 mL), dried over $MgSO_4$, and concentrated in vacuo. The residue was suspended in 500 mL of ether, filtered over Celite, and the filter cake was washed with ether (500 mL). To further remove traces of the catalyst, the filtrate was concentrated to 100 mL and filtered through a membrane filter. The solvent was removed, and chromatography over silica (hexane/ethylacetate 5:1) afforded 12.3 g (81%) of the carboxylic acid **29** as a pale yellow oil. ^{1}H NMR (400 MHz, $CDCl_3$): δ = −0.01 (s, 6H, $Si(CH_3)_2$), 0.86 (s, 9H, $C(CH_3)_3$), 0.88 (d, $^3J_{H,H}$ = 7.1 Hz, 3H, 1'-CH_3), 0.97 (d, $^3J_{H,H}$ = 6.6 Hz, 3H, 5-CH_3), 1.28 (m, 2H), 1.54 (m, 1H), 1.75 (m, 1H), 1.87 (m, 1H), 2.28 (m, 2H), 2.64 (dd, $^3J_{H,H}$ = 7.6, 9.2 Hz, 1H, 1-H), 3.34 (6.9, 10.1 Hz, 1H, 2'-H_a), 3.55 (dd, $^3J_{H,H}$ = 4.8, 10.1 Hz, 1H, 2'-H_b) ppm; ^{13}C NMR (101 MHz, $CDCl_3$): δ = −3.2, 15.6, 16.4, 26.3, 30.5, 32.0, 34.9, 38.2, 40.9, 45.6, 51.6, 67.3, 181.2 ppm; MS *m*/*z* (%): 300 (0) [M^+], 267 (1), 243 (4), 181 (2),123 (70), 81 (44), 75 (100), 73 (30), 55 (20), 41 (27); Anal. calcd for $C_{16}H_{32}O_3Si$: C, 63.95; H, 10.73; found: C, 63.9; H, 10.70%.

(1*S*,2*R*,5*S*)-1-Carboxy-2-(2-*tert*-butyldimethylsilyloxy-(1*S*)-methylethyl)-5-methylyclopentane (29'): Applying the same procedure to 15.7 g (54.8 mmol) of **28'** yielded 11.0 g (67%) of **29'**. The NMR and mass spectra were identical to those of **29**.

(1*R*,2*S*,5*R*)-1-Carboxy-2-(2-hydroxy-(1*R*)-methylethyl)-5-methylcyclopentane (18): To a solution of 5.02 g (16.7 mmol) of the acid **29** in acetonitrile (30 mL) was added 1.7 mL of (40%) aqueous HF at rt, and stirring was continued for 70 min. The

reaction mixture was poured into saturated aqueous $NaHCO_3$ (75 mL) and ether (150 mL) was added. After acidification to pH 4 and separation of the layers, the aqueous phase was extracted with ether (2 x 150 mL). The combined organic extracts were dried over $MgSO_4$ and concentrated in vacuo to give 3.1 g (100%) of the hydroxy acid **18** as a yellow oil which was used without further purification in the next reaction; MS *m*/*z* (%): 186 (0) [M^+], 156 (5), 138 (8), 127 (14), 109 (22), 108 (26), 96 (38), 87 (39), 81 (63), 79 (32), 69 (36), 67 (69), 55 (62), 41 (100), 39 (69); Anal. calcd for $C_{10}H_{18}O_3$: C, 64.49; H, 9.74; found: C, 64.6; H, 9.71%.

(1*S*,2*R*,5*S*)-1-Carboxy-2-(2-hydroxy-(1*S*)-methylethyl)-5-methylcyclopentane (18'): Applying the same method to 5.4 g (18.1 mmol) of **29'** yielded 3.3 g (98%) of **18'**. The mass spectrum was identical to that of **18**.

(4*R*,4a*S*,7*R*,7a*R*)-4,7-Dimethylhexahydrocyclopenta[*c*]pyran-1(3*H*)-one (dihydronepetalactone c): To a solution of 3.1 g (16.7 mmol) of the hydroxy acid **18** in CH_2Cl_2 (1300 mL) were added 3.78 g (18.3 mmol) of DCC and 272 mg (2.2 mmol) of DMAP. After stirring for 1 h at rt, the solvent was removed. The residue was suspended in hexane (375 mL) and filtered. The filter cake was washed with hexane (900 mL), and the filtrate was concentrated. Chromatography over silica (hexane/ethyl acetate 5:1) afforded dihydronepetalactone **c** (1.86 g, 66%) as a pale yellow oil. ^1H NMR (500 MHz, $CDCl_3$): δ = 1.00 (d, $^3J_{H,H}$ = 6.9 Hz, 3H, 4-CH_3), 1.04 (d, $^3J_{H,H}$ = 7.3 Hz, 3H, 7-CH_3), 1.32 (m, 1H, 6-H_a), 1.41 (m, 1H, 5-H_a), 1.75 (m, 1H, 5-H_b), 1.99 (m, 1H, 6-H_b), 2.26 (m, 1H, 4-H_a), 2.32 (m, 1H, 4-H), 2.38 (dd, $^3J_{H,H}$ = 6.8, 13.7 Hz, 1H, 7a-H), 2.51 (m, 1H, 7-H), 4.02 (dd, $^3J_{H,H}$ = 11.3, 11.4 Hz, 1H, 3-H_a), 4.37 (dd, $^3J_{H,H}$ = 4.9, 11.4 Hz, 1H, 3-H_b) ppm; ^{13}C NMR (126 MHz, $CDCl_3$): δ = 11.3 (4-CH_3), 17.9 (7-CH_3), 25.2 (C-5), 29.0 (C-4), 30.9 (C-7), 31.2 (C-6), 38.6 (C-4a), 45.6

(C-7a), 75.9 (C-3), 172.9 (C=O) ppm; MS *m*/*z* (%): 168 (2) [M^+], 126 (13), 113 (80), 110 (16), 95 (42), 82 (26), 81 (100), 69 (47), 68 (30), 67 (76), 55 (37), 53 (37), 41 (95), 39 (78); Anal. calcd for $C_{10}H_{16}O_2$: C, 71.39; H, 9.59; found: C, 71.4; H, 9.50%.

(4*S*,4a*R*,7*S*,7a*S*)-4,7-Dimethylhexahydrocyclopenta[*c*]pyran-1(3*H*)-one (dihydronepetalactone c'): Applying the same procedure to 3.3 g (17.7 mmol) of **18'** yielded 1.9 g (65%) of dihydronepetalactone **c'** as a pale yellow oil. The NMR and mass spectra were identical to those of dihydronepetalactone **c**.

(1*R*,2*R*,5*R*)-1-Acetoxymethyl-2-[(1*R*)-formylethyl]-5-methylcyclopentane (30): To a solution of 1.75 mL (20.1 mmol) of oxalyl chloride in CH_2Cl_2 (100 mL) cooled to −70 °C was added dropwise a solution of 2.86 mL (40.2 mmol) of DMSO in CH_2Cl_2 (5 mL). After 20 min, 2.87 g (13.4 mmol) of the alcohol **26**, dissolved in CH_2Cl_2 (5 mL), was added. Stirring was continued for 1 h before 11.3 mL (81.1 mmol) of triethyl amine was added. The reaction mixture was allowed to slowly warm up to 0 °C over 1 h and then poured onto ice-water (200 mL). The phases were separated, and the aqueous phase was extracted with CH_2Cl_2 (3 x 100 mL). The combined organic extracts were dried over $MgSO_4$ and concentrated. Chromatography over silica (hexane/ethylacetate 8:1, then 4:1) afforded 1.8 g (71%) of the aldehyde **30**. ^{1}H NMR (400 MHz, d_6-DMSO): δ = 0.88 (d, $^3J_{H,H}$ = 5.4 Hz, 3H, 5-CH_3), 1.01 (d, $^3J_{H,H}$ = 5.4 Hz, 3H, 1'-CH_3), 1.28 (m, 4H,), 1.70 (m, 2H), 2.01 (s, 3H, OCH_3), 2.11 (m, 2H), 3.94 (m, 2H, 1-H), 9.61 (d, $^3J_{H,H}$ = 1.0 Hz, 1H, CHO) ppm; ^{13}C NMR (101 MHz, d_6-DMSO): δ = 11.8, 15.0, 21.0, 27.2, 33.5, 35.9, 41.5, 43.6, 50.4, 65.3, 171.2, 204.9 ppm; Anal. calcd for $C_{12}H_{20}O_3$: C, 67.89; H, 9.50; found: C, 67.8; H, 9.47%.

(1*S*,2*S*,5*S*)-1-Acetoxy-2-[(1*S*)-formylethyl]-5-methylcyclopentane (30'): Applying the same oxidation method to 2.54 g (11.9 mmol) of **25'** followed by the same chromatographic purification yielded 1.8 g (79%) of **30'**. Spectroscopic data were identical to those of **30**.

(1*R*,2*R*,5*R*)-1-Acetoxymethyl-2-[(1*R*/*S*)-formylethyl]-5-methylcyclohexane (30 and 30*): To a solution of 1.74 g (8.20 mmol) of the aldehyde **30** in benzene (20 mL), a catalytic amount of *p*-toluenesulfonic acid was added and the reaction mixture was heated under reflux for 6 h. After cooling to rt, the reaction mixture was poured onto an ice-cold aqueous saturated $NaHCO_3$ solution. The phases were separated, and the aqueous phase was extracted with ether (3 x 50 mL). The combined organic extracts were washed with $NaHCO_3$ (50 mL) and brine (50 mL), dried over $MgSO_4$, and concentrated in vacuo to give 1.7 g (96%) of a mixture of diastereoisomers **30** and **30***. ^{1}H NMR analysis confirmed epimerization at the stereogenic center in the side-chain (ratio of **30**:**30*** = 2:3), and the crude mixture of diastereoisomers was used without further purification in the next step. ^{1}H NMR (400 MHz, d_6-DMSO) (representative peaks): δ = 0.98 (d, 3H, 1'*S*-CH_3), 1.01 (d, $^3J_{H,H}$ = 5.4 Hz, 3H, 1'*R*-CH_3), 9.59 (d, 1H, CHO, 1'*S*), 9.61 (d, 1H, CHO, 1'*R*) ppm.

(1*S*,2*S*,5*S*)-1-Aetoxy-2-[(1*R*/*S*)-formylethyl]-5-methylcyclopentane (30'/30*'): Applying the same epimerization reaction to 1.7 g (8.20 mmol) of **30'** yielded 1.7 g (100%) of a 2:3 mixture of **30'** and **30*'**, the corresponding enantiomers of the diastereoisomers **30** and **30***. Analytical data were identical to those of the mixture **30/30***.

(1*R*,2*R*,5*R*)-1-Acetoxymethyl-2-(2-*tert*-butyldimethylsilyloxy-(1*R*/*S*)-methylethyl)-5-methylcyclopentane (27/27*): A solution of 1.53 g (7.22 mmol) of the mixture of the diastereoisomers **30** and **30*** in 19 mL of MeOH was cooled to −20 °C, and 273 mg (7.22 mmol) of sodium borohydride was added in portions. After completion of the reaction as monitored by TLC, the reaction mixture was poured onto a mixture of water (50 mL) and hexane (50 mL). The layers were separated, and the aqueous phase was extracted with ether (2 x 50 mL). The combined organic extracts were washed with brine (50 mL), dried over $MgSO_4$, and concentrated in vacuo to afford a mixture of the diastereoisomeric alcohols **26/26***. MS: *m/z* (%) = 214 (0) [M^+], 136 (5), 123 (30), 121 (20), 95 (86), 81 (46), 67 (32), 43 (100), 41 (38).

The crude mixture of **26/26*** was dissolved in 20 mL DMF, and 1.22 g (18.0 mmol) of imidazole was added at rt. After cooling to −10 °C 1.36 g (9.01 mmol) of TBDMSCl was added in portions, and stirring was continued for 3 h at 0 °C. The reaction mixture was poured onto aqueous $NaHCO_3$ and extracted with hexane (4 x 20 mL). The combined organic extracts were washed with water (40 mL), brine (40 mL), dried over $MgSO_4$, and concentrated in vacuo. Chromatography over silica (hexane/ethyl acetate 40:1 then 15:1) afforded 1.8 g (78%) of a mixture of the diastereoisomers **27/27*** as a yellow oil; ^{1}H NMR (400 MHz, d_6-DMSO): δ = −0.01 (s, 6H, $Si(CH_3)_2$), 0.81 (d, $^3J_{H,H}$ = 5.4 Hz, 3H, 5-CH_3), 0.84 (s, 9H, $C(CH_3)_3$), 0.88 (m, 3H, 1'-CH_3 (*S* and *R*)), 2.02 (s, 3H, OCH_3), 1.15-2.21 (m, 8H), 3.48 (m, 2H, 2'-H), 3.96 (m, 2H, 1-CH_2) ppm; ^{13}C NMR (101 MHz, d_6-DMSO): δ = 12.5, 15.0, 15.1, 18.3, 21.1, 25.9, 26.0, 29.1, 33.8, 33.9, 36.0, 36.6, 38.7, 40.2, 42.4, 43.0, 43.6, 43.9, 65.7, 65.9, 66.6, 67.5, 171.3 ppm (mixture of diastereomers, all signals are listed); MS *m/z* (%): 328 (0) [M^+], 159 (1), 137 (76), 117 (95), 95 (97), 81 (100), 75 (41), 73 (33), 57 (25), 43(40).

(1*S*,2*S*,5*S*)-1-Acetoxy-2-(2-*tert*-butyldimethylsilyloxy-(1*R*/*S*)-methylethyl)-5-methylcyclopentane (27'/27*'): Applying the same reaction sequence to 1.7 g (8.20 mmol) of the mixture of diastereoisomers **30'/30*'** yielded 2.15 g (80%) of **27/27*'**. Spectroscopic data were identical to those of **27/27***.

(1*R*,2*R*,5*R*)-1-Hydroxymethyl-2-(2-*tert-b*utyldimethylsilyloxy-(1*R*/*S*)-methylethyl)-5-methylcyclopentane (28/28*): A solution of 1.52 g (4.63 mmol) of **27/27*** in 5 mL MeOH was added dropwise to 28 mL of a 2 M (56 mmol) solution of KOH in MeOH at 0 °C. After stirring for 2.5 h at rt, the reaction mixture was poured into ice-water (45 mL) and extracted with ether (4 x 50 mL). The combined organic extracts were washed with brine (50 mL), dried over $MgSO_4$, and concentrated in vacuo to give 1.2 g (94%) of a mixture of diastereoisomers **28/28*** as a slightly yellow oil.

^{1}H NMR (400 MHz, d_6-DMSO): δ = −0.03 (s, 6H, $Si(CH_3)_2$), 0.88 (s, 9H, $C(CH_3)_3$), 0.78-0.89 (3 x d, 6H, 5-CH_3 and1'-CH_3 (*R* and *S*)), 1.15–1.95 (m, 8H), 3.45 (m, 4H, 2'-H and 1-CH_2) ppm; ^{13}C NMR (101 MHz, d_6-DMSO): δ = 13.1, 14.2, 15.0, 15.1, 25.9, 28.8, 29.3, 33.9, 34.0, 36.4, 37.3, 38.9, 39.9, 42.9, 44.3, 47.0, 64.1, 64.4, 67.0, 67.5 ppm (mixture of diastereomers, all signals are listed); MS *m*/*z* (%): 286 (0) [M^+], 199 (4), 137 (60), 107 (23), 105 (74), 95 (90), 81 (100), 75 (88), 73 (58), 67 (49), 57 (49), 55 (68), 41 (50).

(1*S*,2*S*,5*S*)-1-Hydroxymethyl-2-(2-*tert-b*utyldimethylsilyloxy-(1*R*/*S*)-methylethyl)-5-methylcyclopentane (28'/28*'): Applying the same method to 2.0 g (6.10 mmol) of **27'/27*'** yielded 1.6 g (93%) of **28'/28*'**. Spectroscopic data were identical to those of **28/28***.

(1*R*,2*S*,5*R*)-1-Carboxy-2-(2-*tert*-butyldimethylsilyloxy-(1*R*/*S*)-methylethyl)-5-methylcyclopentane (29/29*): To a solution of 1.02 g (3.58 mmol) of the mixture of alcohols **28/28*** in a two phase mixture of CCl_4 (7.5 mL), acetonitrile (7.5 mL), and phosphate buffer (11.2 mL, pH 7,) was added 2.30 g (10.6 mmol) of sodium periodate, and stirring was continued for 10 min at rt. After addition of 17.8 mg (6.78 mmol) of ruthenium(III) chloride trihydrate, the resulting mixture was vigorously stirred at rt for 2 h. Subsequently, 25 mL (CH_2Cl_2) and water (20 mL) were added, the layers were separated, and the aqueous phase was extracted with ether (3 x 50 mL). The combined organic extracts were washed with brine (300 mL), dried over $MgSO_4$, and concentrated in vacuo. The residue was suspended in ether (25 mL), filtered over Celite, and the filter cake was washed with 100 mL ether. The solvent was removed, and chromatography over silica (hexane/ethyl acetate 5:1) afforded 742 mg (69%) of the carboxylic acids **29/29*** as a pale yellow oil. ^{1}H NMR (400 MHz, d_6-DMSO): δ = −0.03 (s, 3H, $Si(CH_3)_2$), 0.05 (s, 3H, $Si(CH_3)_2$), 0.88 (s, 9H, $C(CH_3)_3$), 0.88–0.94 (3 x d, 6H, 5-CH_3 and1'-CH_3 (*R* and *S*)), 1.15–1.95 (m, 8H), 3.45 (m, 4H, 2'-H and 1-CH_2) ppm; ^{13}C NMR (101 MHz, d_6-DMSO): δ = 14.2, 15.3, 16.0, 16.2, 28.8, 30.1, 34.2, 34.5, 37.7, 37.8, 40.1, 40.6, 44.4, 45.2, 51.2, 51.7, 67.0, 67.5, 180.1 ppm (mixture of diastereomers, all signals are listed); MS *m*/*z* (%): 300 (0) [M^+], 267 (3), 243 (4), 225 (9), 123 (71), 81 (43), 75 (100), 73 (29), 57 (20), 55 (21), 41 (27).

(1*S*,2*R*,5*S*)-1-Carboxy-2-(2-*tert*-butyldimethylsilyloxy-(1*R*/*S*)-methylethyl)-5-methylcyclopentane (29'/29*'): Applying the same oxidation procedure to 1.04 g (3.64 mmol) of **28'**/**28*'** yielded 865 mg (79%) of **29'**/**29*'**. Spectroscopic data were identical to those of **29**/**29***.

(1*R*,2*S*,5*R*)-1-Carboxy-2-(2-hydroxy-(1*R*/*S*)-methylethyl)-5-methylcyclopentane (18/18*): To a solution of 747 mg (2.42 mmol) of the carboxylic acids **29/29*** in 5 mL acetonitrile was added 0.28 mL 40% aqueous HF at rt, and stirring was continued for 70 min. The reaction mixture was poured into 10 mL of saturated aqueous $NaHCO_3$, and ether (20 mL) was added. After acidification to pH 4 with 1 M HCl and separation of the phases, the aqueous layer was extracted with ether (4 x 20 mL). The combined organic extracts were dried over $MgSO_4$ and concentrated in vacuo to give 441 mg (98%) of the hydroxy acids **18/18*** as a yellow oil, which was used without further purification in the next reaction step; MS *m*/*z* (%): 186 (0) [M^+], 168 (2), 156 (5), 138 (6), 127 (17), 108 (25), 96 (39), 87 (41), 81 (66), 79 (34), 69 (35), 67 (68), 55 (63), 45 (31), 41 (100), 39 (78).

(1*S*,2*R*,5*S*)-1-Carboxy-2-(2-hydroxy-(1*R*/*S*)-methylethyl)-5-methylcyclopentane (18'/18*'): Applying the same procedure to 840 mg (2.72 mmol) of the diastereomeric mixture **29'**/**29*'** afforded 500 mg (99%) of **18'**/**18*'** showing mass spectral data that were identical to those of **18**/**18***. The crude mixture of diastereomers was immediately used for the next step.

(4*S*,4a*S*,7*R*,7a*R*)-4,7-Dimethyl-hexahydrocyclopenta[*c*]pyran-1(3*H*)-one (dihydronepetalactone d): To a solution of 400 mg (2.15 mmol) of the hydroxy acids **18/18*** in CH_2Cl_2 (150 mL) and 694 mg (3.58 mmol) of DCC and DMAP (50 mg, 0.41 mmol) were added. After stirring for 1 h at rt, the solvent was removed. The residue was suspended in hexane (50 mL) and filtered. The filter cake was washed with hexane (3 x 50 mL), and the filtrate was concentrated to yield a 3:2 mixture of the desired product **d** and its previously prepared diastereoisomer **c**. After repetitive column chromatography over silica (hexane/ethyl acetate 5:1), 224 g (62%) pure

(4*R*,4a*R*,7*S*,7a*S*)-dihydronepetalactone **d**, was isolated as a pale yellow oil. ^{1}H NMR (400 MHz, $CDCl_3$): δ = 0.99 (d, $^{3}J_{H,H}$ = 7 Hz, 3H, 4-CH_3), 1.00 (d, $^{3}J_{H,H}$ = 7.2 Hz, 3H, 7-CH_3), 1.20 (m, 1H, 6-H_a), 1.38 (m, 1H, 5-H_a), 1.74 (m, 1H, 5-Hb), 1.85 (m, 1H, 4-H), 2.00 (m, 1H, 6-H_b), 2.15 (m, 1H, 7a-H), 2.29 (m, 1H, 4a-H), 2.50 (m, 1H, 7-H), 3.81 (dd, $^{3}J_{H,H}$ = 11 Hz , 1H, 3-H_a), 4.37 (dd, $^{3}J_{H,H}$ = 5, 11 Hz, 1H, 3-H_b) ppm; ^{13}C NMR (101 MHz, $CDCl_3$): δ = 15.4, 18.1, 28.0, 30.8, 31.6, 35.7, 42.5, 51.4, 76.3, 172.1 ppm; MS *m*/*z* (%): 168 (0) [M+], 153 (4), 139 (3), 126 (13), 113 (84), 110 (14), 95 (41), 81 (100), 67 (74), 55 (41), 41 (94), 39 (77); Anal. calcd for $C_{10}H_{16}O_2$: C, 71.39; H, 9.59; found: C, 71.3; H, 9.50%.

(4*R*,4a*R*,7*S*,7a*S*)-4,7-Dimethylhexahydrocyclopenta[*c*]pyran-1(3*H*)-one (dihydronepetalactone d'): Applying the same procedure to 400 mg of crude **35'/35*'** followed by the same separation process afforded 235 mg (65%) of (4*R*,4a*R*,7*S*,7a*S*)-dihydronepetalactone (**d'**) as a pale oil. The NMR and mass spectra were identical to those of the dihydronepetalactone **d**.
